# Comparative root associated microbial community analysis of *Oreocharis mileensis*, a resurrection plant species with extremely small populations

**DOI:** 10.3389/fmicb.2025.1692695

**Published:** 2025-11-04

**Authors:** Temur Asatulloev, Ziyoviddin Yusupov, Lei Cai, Qiuping Chen, Bishal Gurung, Komiljon Sh. Tojibaev, Weibang Sun

**Affiliations:** ^1^Yunnan Key Laboratory for Integrative Conservation of Plant Species With Extremely Small Populations, Kunming Institute of Botany, Chinese Academy of Sciences, Kunming, China; ^2^Institute of Botany, Academy of Sciences, Tashkent, Uzbekistan; ^3^University of Chinese Academy of Sciences, Beijing, China; ^4^Yunnan Key Laboratory for Wild Plant Resources, Department of Economic Plants and Biotechnology, Kunming Institutes of Botany, Chinese Academy of Sciences, Kunming, China

**Keywords:** *Oreocharis mileensis*, comparative microbiome diversity, drought stress, conservation, core taxa

## Abstract

Plants dynamically interact with their microbiomes through phytohormonal signaling and defense responses, shaping microbial diversity and ecosystem function. While resurrection plants host growth-promoting and drought associated microbes, prior studies on different resurrection plants have been limited to localized sampling, potentially underestimating microbial diversity. We analyzed bacterial and fungal communities across five populations of *Oreocharis mileensis*, a resurrection plant, during hydrated and dehydrated states to examine population-level microbiome differences or affinity, identify microorganisms that may assist during plant desiccation, and assess their conservation across populations. We found that microbial composition was strongly influenced by compartment (bulk soil, rhizosphere, and endosphere) but exhibited only moderate drought-induced changes, suggesting that O. mileensis maintains a stable microbiome under stress. Core phyla (e.g., Proteobacteria, Actinobacteriota, Ascomycota) were conserved across populations, but genus-level core taxa varied relatively between populations, reflecting niche specialization and host genotype. Drought increased bacterial alpha diversity while reducing beta diversity, indicating homogenization driven by stress-tolerant taxa such as Actinobacteriota. Fungal responses differed, with increased beta diversity suggesting drought-enhanced compositional turnover. Key bacterial genera (e.g., Burkholderia-Caballeronia-Paraburkholderia, Bacillus, Rhizobium) dominated hydrated states, while drought enriched Actinobacteria (e.g., Microlunatus, Rubrobacter) and other drought-resistant taxa. Fungal communities shifted from saprotroph-dominated hydrated states to symbiotic taxa (e.g., Paraboeremia, Helotiales) under drought conditions. Functional profiling revealed compartment-specific metabolic specialization, with drought enriching stress-response pathways (e.g., secondary metabolite biosynthesis, signal transduction). These findings demonstrate that *O. mileensis* microbiomes are structured by compartmental filtering and exhibit drought-driven functional plasticity, with conserved stress-adapted taxa potentially supporting host resilience. Overall, this study expands our understanding of microbiome assembly in resurrection plants and highlights candidate microbes for microbiome engineering to enhance crop stress tolerance.

## Introduction

A small but remarkable subset of angiosperms has evolved the ability to survive extreme desiccation, recovering fully upon rehydration. Known as resurrection plants, these species can tolerate severe water loss, maintaining cellular integrity and metabolic function even at <10% relative water content (Farrant and Moore, 2011). To date, approximately 300 angiosperm resurrection plant species have been discovered (Gaff and Oliver, 2013), with the Velloziaceae family containing most of these species. Their extraordinary resilience makes them invaluable models for studying drought adaptation, with potential applications in crop improvement and ecological restoration. While extensive research has elucidated physiological and molecular mechanisms underlying their desiccation tolerance, the role of plant-associated microbiomes, particularly bacteria and fungi, in facilitating this resilience remains largely unexplored. Recent studies on resurrection plants like *Boea hygrometrica* (Sun et al., 2024) and *Myrothamnus flabellifolia* (Tebele et al., 2024, 2025) highlight the importance of microbial communities in stress mitigation, nutrient acquisition, and growth promotion. However, these investigations have been limited to single populations or locations, leaving a critical gap: whether microbial associations are conserved across diverse ecological populations of the same species or vary with environmental context.

The endangered resurrection plant *Oreocharis mileensis* (Gesneriaceae) offers an ideal system to address this question. Endemic to karst limestone habitats in southwestern China, *O. mileensis* survives prolonged drought and rapidly resumes growth upon rehydration (Qin et al., 2017). Unlike prior studies on widespread resurrection species (e.g., *Craterostigma plantagineum*), this study examines bulk soil, rhizosphere, and endosphere microbiomes across five distinct populations of *O. mileensis*, providing the first comparative analysis of microbial contributions to desiccation tolerance in fragmented ecological settings. As a Plant Species with Extremely Small Populations (PSESP) (Sun, 2013; Sun et al., 2019), *O. mileensis* faces severe threats from habitat destruction and climate change, necessitating urgent research into ecological factors, including microbial symbionts, that sustain its survival.

The rhizosphere and endosphere microbiomes are of particular interest due to their direct influence on plant health. Drought-adaptive microbes, such as plant-growth-promoting rhizobacteria (PGPR) and mycorrhizal fungi, can enhance water retention, produce stress-alleviating phytohormones, and improve nutrient uptake (Vurukonda et al., 2016; Naylor et al., 2017). Identifying core microbiome members shared across *O. mileensis* populations could reveal conserved mechanisms of desiccation tolerance, while population-specific microbial signatures may reflect local adaptations to environmental pressures. Such insights are critical for conservation, as microbial diversity loss from habitat degradation could further imperil *O. mileensis* by disrupting these vital interactions.

Supporting this approach, studies on other endangered plants demonstrate the pivotal role of microbiomes in species survival. For example, reintroducing native soil microbes has improved establishment rates in restoration projects (Parker et al., 2006), and resurrection plants like *B. hygrometrica* harbor specialized microbes (e.g., *Sphingosinicella, Plectosphaerella*, and Ceratobasidiaceae fungi) that enhance desiccation tolerance (Sun et al., 2024). For *O. mileensis*, microbiome-assisted propagation could bolster ex situ conservation efforts, but only if key microbial partners are first identified. Despite this potential, no study has yet characterized the bacterial and fungal communities associated with *O. mileensis* or assessed their variability across its restricted range. Closing this knowledge gap is essential to develop targeted strategies that preserve both the plant and its microbial allies, ensuring resilience in the face of escalating environmental threats.

## Materials and methods

### Experimental design and sample collection

**Figure 1:**
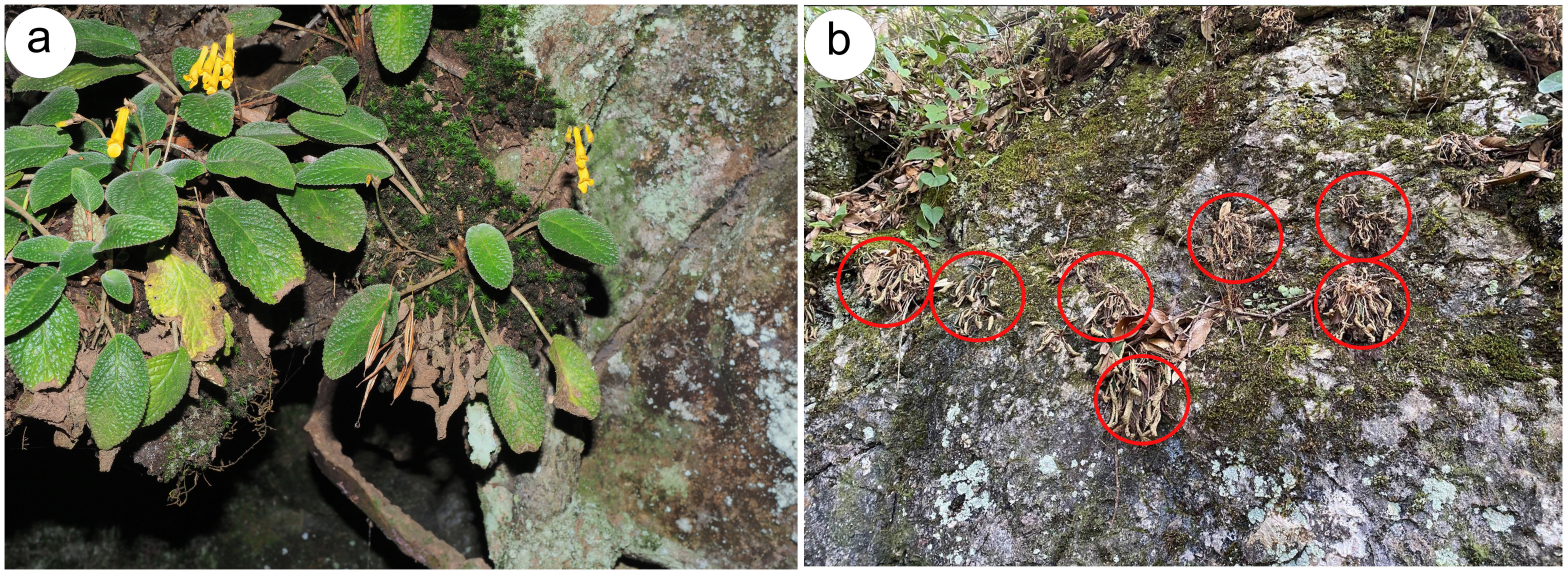
Sample collection. **(a, b)** Photographs of *O. mileensis* plants in the hydrated **(a)** and dehydrated **(b)** states in their natural habitat.

The plant and soil samples were collected from five distinct populations of *O. mileensis* to cover the whole distribution range and landscape variation: Xiaopingzhang, E'Jia Town, Shuangbai County (EJIA population, 24°20′28.6476″ N, 101°19′2.84″ E); Gubai, Wushan Town, Mile City (GB population, 24°16′4.88″ N, 103°15′9.93″ E); Xiaotuantian, Guishan Town, Shilin County (GS population 24°37′16.34″ N, 103°33′38.69″ E); Qian Tao, Huaxi District, Guiyang City (QT population, 26°18′12.6252″ N, 106°44′17.484″ E); and Bai Gelong, Matang Town, Wenshan City (WS population, 23°27′25.56″ N, 104°10′2.37″ E) in 2024. The sampling locations are shown in Supplementary Figure S1a. Two states of *O. mileensis* plants were used in this study: the hydrated state (H) and the subsequent dehydrated state (D) after a dry period of more than 3 weeks without rainfall (Figures 1a, b). The whole plant and its surrounding native soil were harvested from five independent naturally growing populations within 2 m^2^. A total of 20 individual plant and soil samples (four sub-samples per population as biological replicates) were collected from hydrated plants, and another set of samples was collected from dehydrated plants. Unseparated plant and soil samples were immediately placed in sterile bags and transported to the laboratory on ice.

Loose soil attached to the roots was removed by vigorous shaking and classified as bulk soil (B), which was used for microbial DNA analysis and relative water content (RWC) determination. Rhizosphere soil (R) was gently brushed off the root surface for water content determination. R and root endosphere (E) samples were isolated using a modified protocol based on Edwards et al. (2018). For rhizosphere separation, roots with firmly attached soil were vortexed three times in phosphate-buffered saline (PBS). The resulting suspension was centrifuged to pellet microbial cells, which were collected as the R fraction. To isolate the endosphere compartment, roots were subsequently sonicated in PBS until visually free of soil particles, then surface-sterilized through sequential immersion in:

- 75% ethanol (1 min)
- 10% sodium hypochlorite (3 min)
- Three rinses with sterile distilled water

The sterilized roots were processed as E samples for endosphere microbial DNA extraction.

### DNA extraction, amplicon sequencing, and OTU assignment

Genomic DNA was extracted from all samples using a CTAB/SDS protocol. DNA purity and concentration were assessed using NanoDrop 2000 spectrophotometry (Thermo Fisher Scientific) and agarose gel electrophoresis. For bacterial community analysis, the V5–V7 region of 16S rRNA was amplified using primers 799F/1193R, while the fungal ITS region was targeted with primers ITS1F/ITS2R. PCR reactions (15 μL in volume) contained Phusion High-Fidelity Master Mix (New England Biolabs), 0.2 μM of each primer, and 10 ng template DNA, with cycling parameters as follows: 98 °C (1 min); 30 cycles of 98 °C (10 s), 50 °C (30 s), 72 °C (30 s); and a final extension at 72 °C (5 min). Purified amplicons were quantified using Qubit and sequenced on Illumina PE250. Paired-end sequencing reads were merged using FLASH (Magoč and Salzberg, 2011) and quality-filtered with fastp (Chen et al., 2018) to generate high-quality clean tags. For bacterial 16S sequences, chimeras were detected and removed through alignment against the SILVA database (Quast et al., 2012), while fungal ITS sequences were processed using the UNITE (Abarenkov et al., 2024) database. Quality-filtered sequences were clustered into operational taxonomic units (OTUs) at 97% similarity using the UPARSE algorithm (Edgar, 2013), with taxonomic classification performed using Mothur (Schloss et al., 2009) for bacteria and BLAST (Camacho et al., 2009) for fungi against their respective reference databases.

### RWC determination

To measure relative water content (RWC), leaves from each plant were first weighed to obtain the fresh weight (FW). The samples were then soaked in distilled water for 6 h at room temperature in the dark to reach full hydration. After this period, the turgid weight (TW) was recorded. The samples were then rapidly dried in an oven at 105 °C for 1 h, followed by drying at 65 °C until a constant weight was achieved to determine the dry weight (DW). RWC was calculated using the formula: RWC (%) = [(FW – DW)/(TW – DW)] × 100. Soil water content (SWC) was measured using the oven-drying method, where samples were dried at 105 °C until a stable weight was reached. Statistical analyses of plant physiological parameters and SWC were conducted using independent samples *t*-tests for comparisons between two groups in R (v4.0.3). The plant leaves, bulk, and rhizosphere soils were measured to determine significant water loss. We determined that all group pairs (hydrated leaf vs. dehydrated leaf, hydrated bulk soil vs. dehydrated bulk soil, hydrated rhizosphere soil vs. dehydrated rhizosphere soil) within each population showed significant water loss (independent *t*-tests, *p* < 0.05 to 0.001; Supplementary Figure S1b).

### Statistical analysis

We determined core taxa according to prevalence (if a particular taxon occurred in 90% of samples or more) and abundance (a particular taxon occurred in 1% abundance or more across all samples) when considering all populations together. However, within a single population, we considered the prevalence, abundance, and ubiquity of a particular taxon. Microbial alpha diversity was calculated in R (v4.4.3), including richness estimators (Sobs, Chao1, ACE), diversity indices (Shannon, Simpson), and coverage metrics. In contrast, beta diversity was assessed using weighted/unweighted UniFrac distances visualized through principal coordinate analysis (PCoA) and hierarchical clustering (UPGMA). Functional potential was predicted using PICRUSt2 (v2.3.0) (Douglas et al., 2020) with KEGG/COG/MetaCyc (Caspi et al., 2014; Galperin et al., 2025; Kanehisa et al., 2025) databases for bacteria and FUNGuild (v1.0) (Nguyen et al., 2016) for fungal ecological guilds. These analyses were conducted by pooling samples from all five populations, rather than analyzing each population separately. This approach was chosen to focus on the overall functional shifts associated with compartments (bulk soil, rhizosphere, and endosphere) and moisture conditions (drought vs. hydrated), which were the primary factors of interest in this study. Statistical analyses included Wilcoxon rank-sum tests for two-group comparisons and Kruskal–Wallis tests for three or more groups, with significance thresholds set at *p* < 0.05 for diversity and functional/species differences analyses. Differential abundance analyses were conducted with the microeco package (Liu et al., 2021) in R. All statistical analyses and visualizations were performed using R software.

## Results

### Overview of the OTU data

**Figure 2:**
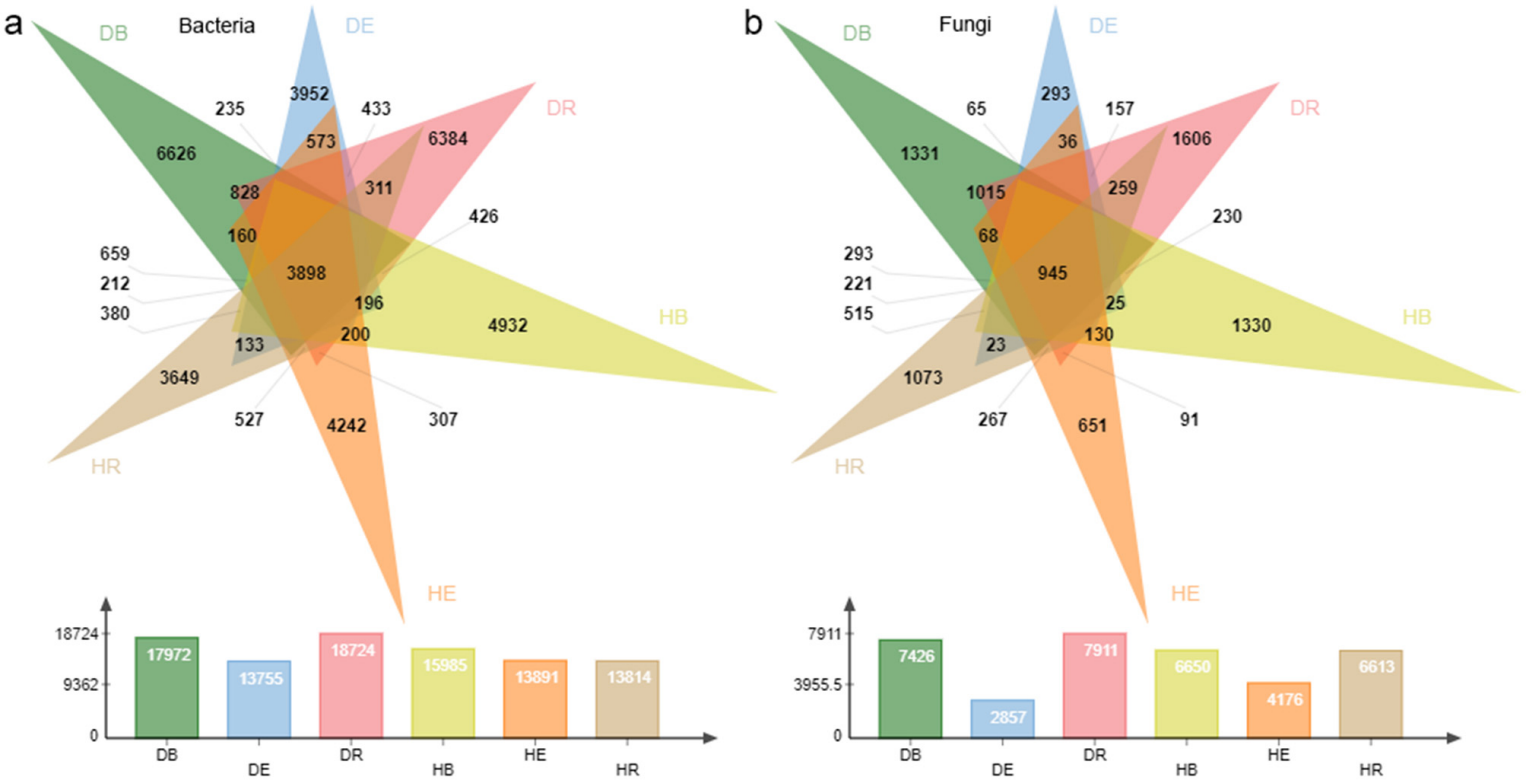
Comparison of operational taxonomic units in the compartments across hydrated and dehydrated states. **(a, b)** Venn diagrams showing the overlap of bacterial **(a)** and fungal **(b)** OTUs among samples. The bars in the lower panel indicate the total number of OTUs within each compartment in the dehydrated and rehydrated states. DB, dehydrated bulk soil; DE, dehydrated root endosphere; DR, dehydrated rhizosphere; HB, hydrated bulk soil; HE, hydrated root endosphere; HR, hydrated rhizosphere.

To comprehensively grasp the compositions and drought response of *O. mileensis* plant-associated endophytic and exogenous microorganisms, samples from B, R, and E under hydrated (H) and dehydrated (D) conditions were selected and subjected to high-throughput 16S rRNA and ITS amplicon sequencing. A total of 47,333 bacterial OTUs and 15,101 fungal OTUs were generated from the quality-filtered 16S rRNA and ITS sequencing reads across all samples, respectively. The flattening rarefaction curves of both bacterial and fungal communities indicated sufficient sampling depth (Supplementary Figures S1c, d). A total of 3,898 bacterial OTUs and 945 fungal OTUs were shared across all three compartments of both H (hydrated) and D (dehydrated) groups when all five populations were considered together (Figures 2a, b). The exogenous (DB and DR) bacterial communities from the D group had a greater number of unique OTUs than those in the endophytic (DE) compartments (Figure 2a). In general, both bacterial and fungal communities had a greater number of unique OTUs in the D group than in the H group (but DE had fewer unique OTUs than HE). Notably, the number of unique bacterial and fungal OTUs in the D group was greater in DR than in HR of the H group at the single-population level (except the QT population; Supplementary Figures S2, S3).

### Microbial community compositions of *O. mileensis*

**Figure 3:**
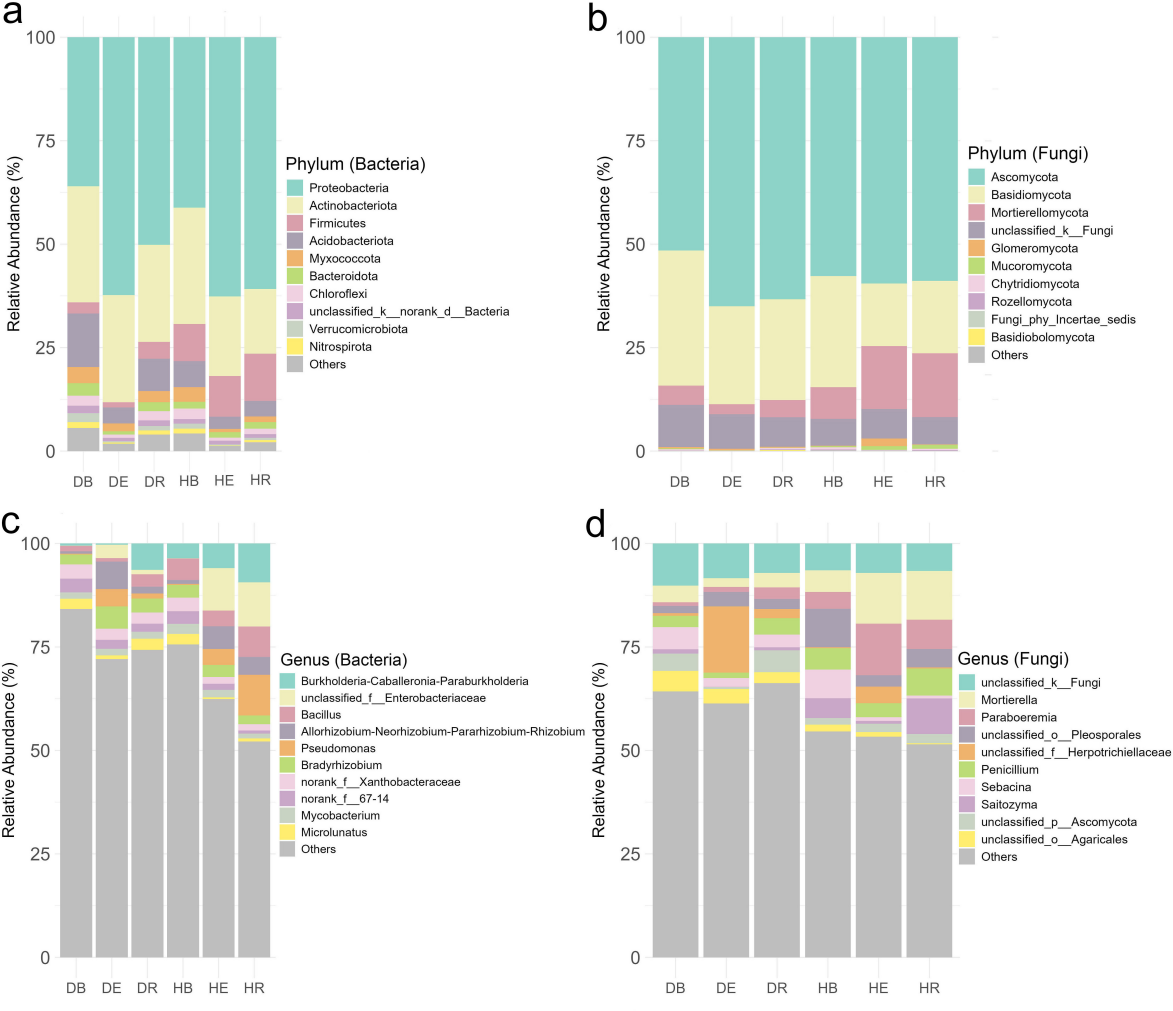
Relative abundance of bacterial **(a, c)** and fungal **(b, d)** community composition. **(a, b)** Show the genus level, while **(c, d)** show the phylum level. DB, dehydrated bulk soil; DE, dehydrated root endosphere; DR, dehydrated rhizosphere; HB, hydrated bulk soil; HE, hydrated root endosphere; HR, hydrated rhizosphere.

Taxonomic analysis identified 2,961 bacterial species spanning 35 phyla and 1,183 genera, alongside 3,321 fungal species across 18 phyla and 1,716 genera in both H and D groups combined when all populations were analyzed together (Supplementary Tables S1, S2). At the genus level, core bacterial taxa included Burkholderia-Caballeronia-Paraburkholderia (4.58%) and Bacillus (3.7%), while core fungi were dominated by unclassified Fungi (7.6%) and Mortierella (6.5%) (Figures 3c, d; Supplementary Table S5). At the genus level, core bacterial taxa included *Burkholderia-Caballeronia-Paraburkholderia* (4.58%) and *Bacillus* (3.7%), while core fungi were dominated by unclassified Fungi (7.6%) and *Mortierella* (6.5%) (Figures 3a, b; Supplementary Table S5). The bacterial and fungal communities associated with different populations of *O. mileensis* exhibited a consistent pattern, characterized by high diversity and underpinned by a shared core of abundant taxa at the genus level (Supplementary Figures S4–S7; Supplementary Table S5). This common structure was reflected at the genus level, where each population showed distinct dominant taxa coexisting with a ubiquitous core. For bacteria, the core ubiquitous genera included *Burkholderia-Caballeronia-Paraburkholderia, Bacillus, Allorhizobium-Neorhizobium-Pararhizobium-Rhizobium, Bradyrhizobium, Pseudomonas, Mycobacterium*, and *Microlunatus*. Population-specific variations were observed, featuring enrichments of *Burkholderia-Caballeronia-Paraburkholderia* in EJIA (11.35%) and QT (5.55%), *Bacillus* in GB (8.30%), and unclassified Enterobacteriaceae in GS (6.62%) and WS (7.77%). The fungal communities displayed a parallel pattern, with abundant ubiquitous genera (including *Mortierella, Penicillium*, unclassified Herpotrichiellaceae, and *Paraboeremia*) identified across populations. Notable specializations included a high abundance of *Mortierella* in GS (13.99%), and the dominance of unclassified Pleosporales (16.00%) and *Paraboeremia* (13.65%) in WS.

### Microbial diversity patterns across soil–root compartments

**Figure 4:**
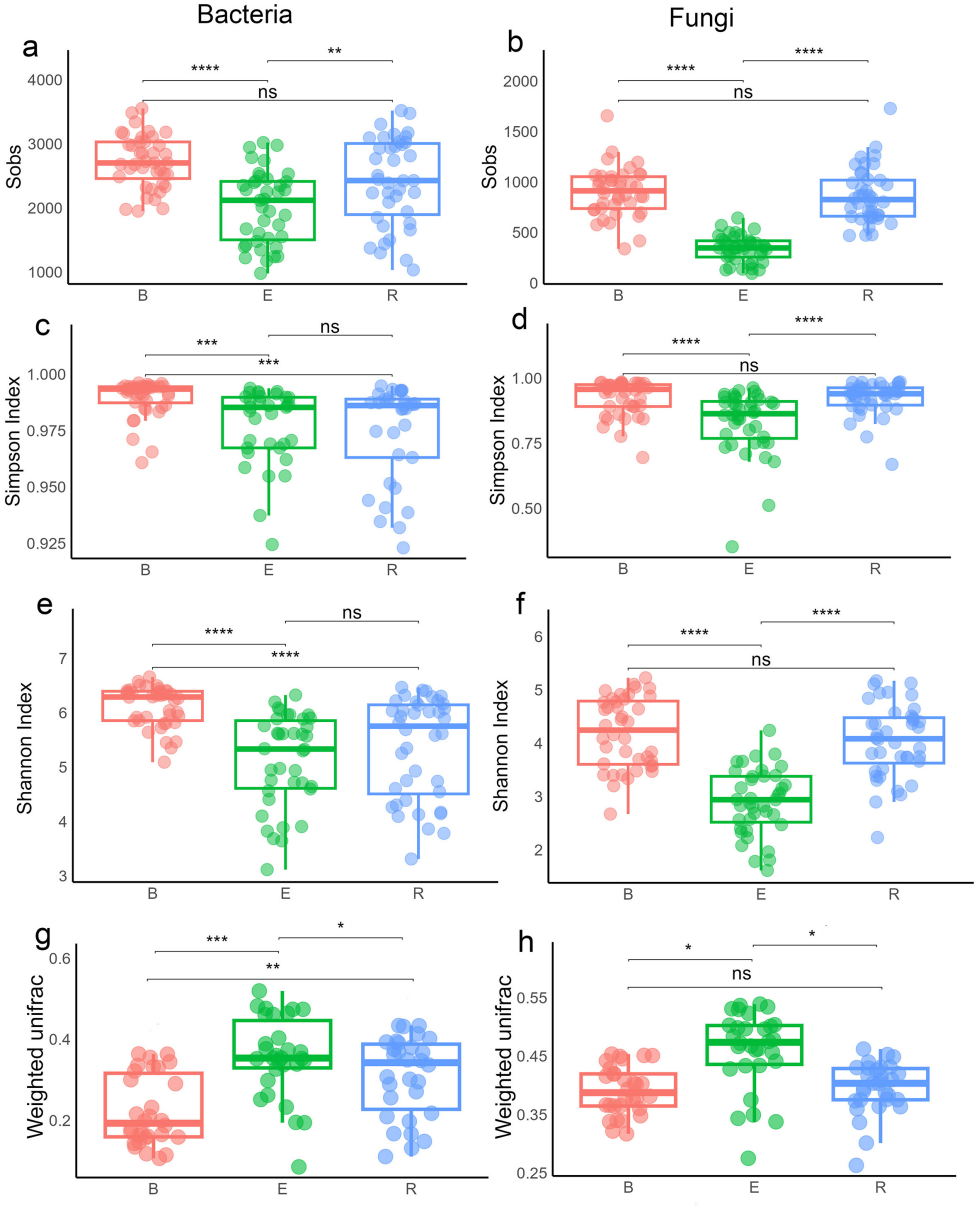
Alpha and beta diversity of microbial communities across three compartments. **(a, b)** Sobs diversity index of bacterial **(a)** and fungal **(b)** communities. **(c, d)** Simpson diversity index of bacterial **(c)** and fungal **(d)** communities. **(e, f)** Shannon diversity index of bacterial **(e)** and fungal **(f)** communities. **(g, h)** Beta diversity of bacterial **(g)** and fungal **(h)** communities based on the weighted UniFrac distance matrices. B, bulk soil; R, rhizosphere soil; E, root endosphere. Statistical differences were determined using the Wilcoxon rank-sum test. Significance codes (indicated with asterisks): *p < 0.05, **p < 0.01, **p < 0.001, and ****p < 0.0001. Boxes sharing the same letters in **(g, h)** are not significantly different.

Analysis of microbial communities revealed distinct compartmentalization between bacteria and fungi across all diversity metrics when all populations were considered together. For species richness (Sobs), both groups were higher in B than E (*p* < 0.0001; Figures 4a, b; Supplementary Table S6). R did not differ from B (*p* > 0.05) but remained distinct from E (bacteria: *p* < 0.01; fungi: *p* < 0.0001). Chao1 showed E differed significantly from both B and R (*p* < 0.01 to 0.0001), while R vs. B was non-significant. ACE was consistent with this, with stronger fungal effects (*p* < 0.0001) than bacterial (*p* < 0.01 to 0.0001).

Simpson index indicated greater bacterial evenness in B than in R or E, with no difference between E and R. In fungi, evenness in B differed from that in E (*p* < 0.0001) but not in R, while E and R were distinct (Figures 4c, d). Shannon index indicated that bacterial diversity was higher in B than in R or E (both *p* < 0.0001), whereas fungal diversity was higher in B than in E (*p* < 0.0001) and in R (*p* < 0.05) (Figures 4e, f).

Beta diversity further supported compartmentalization. Please change this sentence to: Weighted UniFrac showed significant differentiation for bacteria (E and B: *p* < 0.001; R and E: *p* < 0.05; B and R: *p* < 0.01) and fungi (only E is different from other compartments) (Figures 4g, h). Unweighted UniFrac confirmed stronger bacterial signals (all pairwise compartments *p* < 0.0001) than fungal (E vs. B, R vs. E: *p* < 0.0001) (Supplementary Table S6). At the single-population level, alpha and beta diversity patterns were similar but weaker (Supplementary Figures S8–S11; Supplementary Table S7).

Ordination of bacterial and fungal communities based on OTU and species abundance matrices revealed no significant separation of the B, R, and E compartments of bacterial and fungal communities by PCA and PLS-DA when all populations were analyzed together (Supplementary Figures S12, S13). At the single-population level, bacterial compartments were generally separated by PCA (except in the EJIA population), explaining at least 20.94% of variance in PC1 and 12.29% in PC2, though with minor overlaps (Supplementary Figure S12). For fungi, E separated from B and R but with overlaps, while B and R overlapped extensively (Supplementary Figure S13). PLS-DA of bacteria further distinguished E from B and R, which remained overlapping (Supplementary Figure S12). In fungi, only E separated from B and R in EJIA, while all compartments overlapped in other populations (Supplementary Figure S13).

### Differential abundance of microorganisms across compartments

**Figure 5:**
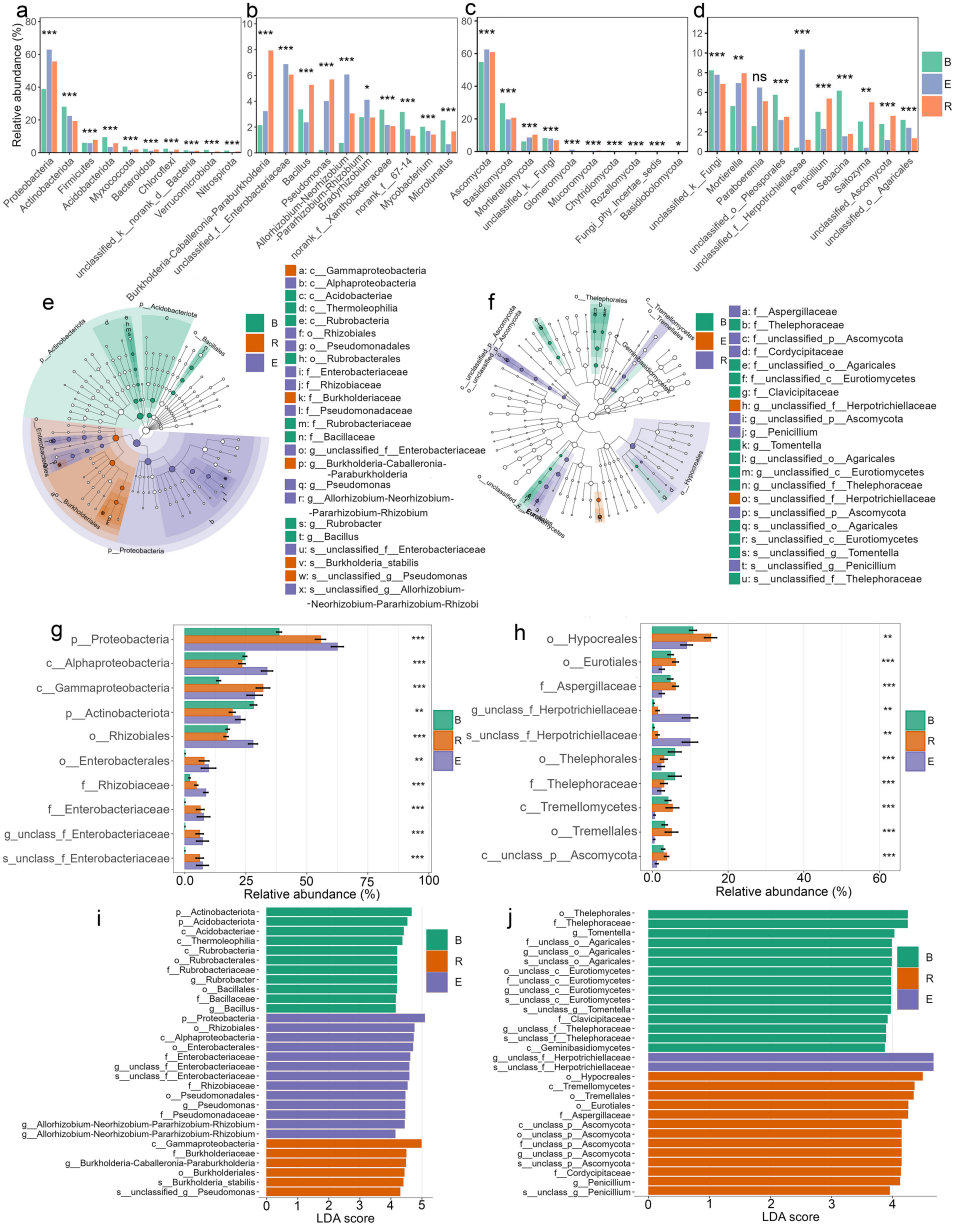
Differential microbial abundance across three compartments (B, bulk soil; R, rhizosphere; E, endosphere). **(a–d)** Relative abundance of dominant bacterial **(a, b)** and fungal **(c, d)** taxa at the phylum **(a, c)** and genus **(b, d)** levels. **(e, f)** Differentially abundant bacterial **(e)** and fungal **(f)** taxa (phylum to genus). **(g, h)** Mean relative abundance (±SEM) of selected bacterial **(g)** and fungal **(h)** taxa (phylum to species). **(i, j)** Top 30 bacterial **(i)** and fungal **(j)** taxa ranked by LDA score (log10). *All differential analyses were performed using LEfSe (α <0.01, LDA > 3). Significance codes (indicated with asterisks): *p < 0.05, **p < 0.01, ***p < 0.001, ****p < 0.0001.

There were significant abundance differences in all of the dominant (top 10) bacterial phyla (10/10) and genera (10/10) when all populations were analyzed together, regardless of hydration status (*p* < 0.05 to <0.001; Figures 5a, b). The microbial community composition varied markedly across compartments, with Proteobacteria dominating in E (62.5%) compared to B (38.7%) and R (55.7%). Actinobacteriota were more abundant in B (28.1%) than in E (22.4%) or R (19.4%), while Firmicutes were more prevalent in R (7.9%) than in B (6.0%) or E (5.7%). Several phyla, including Acidobacteriota, Chloroflexi, Myxococcota, and Nitrospirota, exhibited consistently lower relative abundances in E than in B and R, suggesting compartment-specific selection pressures. Minor phyla such as Bacteroidota and Verrucomicrobiota also had lower representation in E, further highlighting distinct microbial structuring across compartments. Furthermore, the analysis revealed both shared and population-specific phylum distributions. All five populations contained Proteobacteria, Actinobacteriota, Firmicutes, and Acidobacteriota as core components of their microbial communities (Supplementary Figure S14). However, their relative prominence varied substantially between populations. Several phyla showed restricted distributions, with Entotheonellaeota present only in GB, QT, and WS populations, while Nitrospirota was unique to QT and WS. GS showed near-exclusive dominance by Proteobacteria with minimal representation of other phyla, whereas WS displayed enhanced Firmicutes alongside the core phyla. The genus-level composition revealed strong compartment-specific trends (Figure 5b) when all populations were considered together. *Burkholderia-Caballeronia-Paraburkholderia* was highly enriched in R (7.9%) compared to E (3.2%) and B (2.1%), while *Allorhizobium-Neorhizobium-Pararhizobium-Rhizobium* showed higher abundance in E (6.1%) than in B (0.8%) or R (3.0%). *Bacillus* and *Pseudomonas* were notably more prevalent in R (5.3% and 5.7%, respectively) than in other compartments. Conversely, *norank_f__67-14* and *Microlunatus* were more abundant in B (3.2% and 2.5%, respectively) than in E and R. unclassified Enterobacteriaceae dominated in E (6.9%) and R (6.1%) but was nearly absent in B (0.06%). *Bradyrhizobium* and *Mycobacterium* exhibited moderate representation across compartments, with *Bradyrhizobium* peaking in E (4.1%) and *Mycobacterium* in B (2.0%). Single-population level analyses revealed both shared distributions and population-specific specialization patterns (Supplementary Figure S14). Certain genera, such as *Bradyrhizobium* was consistently abundant in four populations (*p* > 0.05, except EJIA) and *Burkholderia-Caballeronia-Paraburkholderia*, was abundant in R in most populations (*p* < 0.001), while *Allorhizobium-Neorhizobium-Pararhizobium-Rhizobium* dominated E in four populations (*p* < 0.01 to <0.001), whereas others showed restricted distributions. *Mycobacterium* occurred in GB, GS, and QT populations and was present in B (abundant), R, and E compartments (*p* < 0.05 to <0.001). The EJIA population displayed the most distinct composition, featuring unique dominance of *Acidothermus* in bulk soil and *Lactococcus* in the endosphere (*p* < 0.001), genera that were rare in other populations. Population-specific patterns emerged clearly in bulk soils: EJIA maintained *norank_f__Micropepsaceae* abundant in B compartment but also present in R and E, while GS featured *norank_f__67-14*. The endosphere revealed specialized associations, with *unclassified_f__Enterobacteriaceae* particularly abundant in GB, GS, and WS populations' rhizosphere and endosphere (*p* < 0.001, abundant in endosphere). Notable low-abundance genera further differentiated populations; for instance, *Pseudonocardia* occurred exclusively in GS, QT, and WS (abundant in endosphere, *p* >0.05 to <0.001).

There were significant abundance differences across three compartments in all of the dominant (top 10) fungal phyla (10/10) and genera (9/10) when all populations are analyzed together (*p* > 0.05 to <0.001; Figures 5c, d). Ascomycota represented the dominant phylum, particularly in R and E compartments, accounting for 60.9% and 62.4% of sequences, respectively, compared to 54.8% in bulk soil. In contrast, Basidiomycota showed an inverse distribution pattern, being significantly more abundant in bulk soil than in the R or E compartments (*p* < 0.001). Mortierellomycota and Glomeromycota were consistently enriched in the E compartment (*p* < 0.001).

When examining individual populations, compartment-specific patterns became more nuanced (Supplementary Figure S14). Ascomycota maintained their predominance in endosphere communities across all populations, ranging from 62.4% to 76.9% relative abundance, while their representation in bulk soil and root compartments showed greater variability (42.9–74.1% and 46.9–78.5%, respectively). The population-level analysis revealed that Mortierellomycota's root enrichment was particularly pronounced in EJIA, GB, and GS populations, with significant abundance in R and E compartments compared to B (*p* < 0.01 to <0.001), though this pattern was not statistically significant in other populations. Glomeromycota's endosphere association remained consistent across most populations (*p* < 0.001), with the exception of EJIA, where no significant compartment preference was observed. Rare phyla displayed distinct population-specific distributions, such as Kickxellomycota, which appeared with significant abundance only in B compartments of GB and WS populations (*p* < 0.05), and Olpidiomycota, which was restricted to EJIA and GS with significant but low abundance in R compartments (*p* < 0.05). Similar compartment-driven patterns were observed at the genus level, as seen in the bacterial community, where significant abundance differences were detected for most dominant taxa (9/10 genera; *p* > 0.05 to *p* < 0.001), further emphasizing the strong influence of compartment niche on fungal community structure when all populations were considered together (Figure 5d). Top abundant genera were consisted of unclassified fungi (*p* < 0.001; abundant in B: 8.2%, but also present in R and E), *Mortierella* (*p* < 0.01; abundant in R: 7.95%, but also present in E), *Paraboeremia* (*p* > 0.05; abundant in E: 6.5%), unclassified Pleosporales (*p* < 0.001; abundant in B: 5.7%), unclassified Herpotrichiellaceae (*p* < 0.001; abundant in E: 10.3%), *Penicillium* (*p* < 0.001; abundant in R: 5.3%), *Sebacina* (*p* < 0.001; abundant in B: 6.1%), *Saitozyma* (*p* < 0.01; abundant in R: 4.9%), unclassified Ascomycota (*p* < 0.001; abundant in R: 3.6%), and unclassified Agaricales (*p* < 0.001; abundant in B: 3.2%). When examining individual populations, compartment-specific fungal distributions revealed both conserved and population-unique patterns (Supplementary Figure S14). GB populations exhibited distinct niche partitioning, with *Paraboeremia* and *Dactylonectria* dominating the endosphere (*p* > 0.05), while *Penicillium* (*p* < 0.05) and *Thelonectria* (*p* > 0.05) showed rhizosphere preference. *Mortierella* demonstrated compartment-dependent associations across populations, with significant rhizosphere enrichment in GS (*p* > 0.05) and EJIA (*p* < 0.05), yet bulk soil preference in GB (4.5%). The QT population displayed unique taxonomic signatures, with *Alfoldia* and unclassified Nectriaceae restricted to endosphere niches, while *Saitozyma* showed exclusive rhizosphere abundance (*p* > 0.05). WS population was characterized by *Paraboeremia* (*p* > 0.05) and unclassified Herpotrichiellaceae in the endosphere (*p* < 0.001), contrasting with *Hygrocybe* and *Penicillium* in the rhizosphere (*p* > 0.05 and *p* < 0.001, respectively). Universal taxa exhibited niche-driven abundance shifts. Unclassified fungi were present in all populations, peaking in B, R, and E compartments depending on the specific population. Population-restricted distributions were evident for rare taxa: *Sebacina* occurred in QT and GS (*p* < 0.05 and *p* < 0.001; abundant in B than R and E) and *Tomentella* in GS and GB (*p* < 0.001; abundant in B than R and E), marking population-specific bulk soil indicators. The rhizosphere niche consistently selected for *Saitozyma* in QT and EJIA (*p* > 0.05), whereas endosphere communities preferentially hosted host-adapted taxa, such as unclassified Hyaloscyphaceae, unclassified Capnodiales, and *Trechispora* in EJIA, and unclassified Herpotrichiellaceae in WS, QT, and GS during drought (*p* < 0.05 to 0.001).

The LefSe analysis, from phylum to genus, showed that 296 bacterial genera within 23 phyla and 141 fungal genera within 4 phyla determined the dissimilarities among the three compartments (LDA > 2 and *p* < 0.05 to 0.001; Figures 5e, f). The dominant phylum of the bacterial community in B was Actinobacteriota, while Proteobacteriota was dominant in E and R. For the fungal community, Thelephorales was dominant in B, Hypocreales in R, and Herpotrichiellaceae in E (Figures 5g–j). Additionally, a total of 570 bacterial species and 110 fungal species were significantly differentially enriched across all compartments (LDA > 2, *p* < 0.05 to <0.001).

### Impact of drought stress on soil microbial community structure

**Figure 6:**
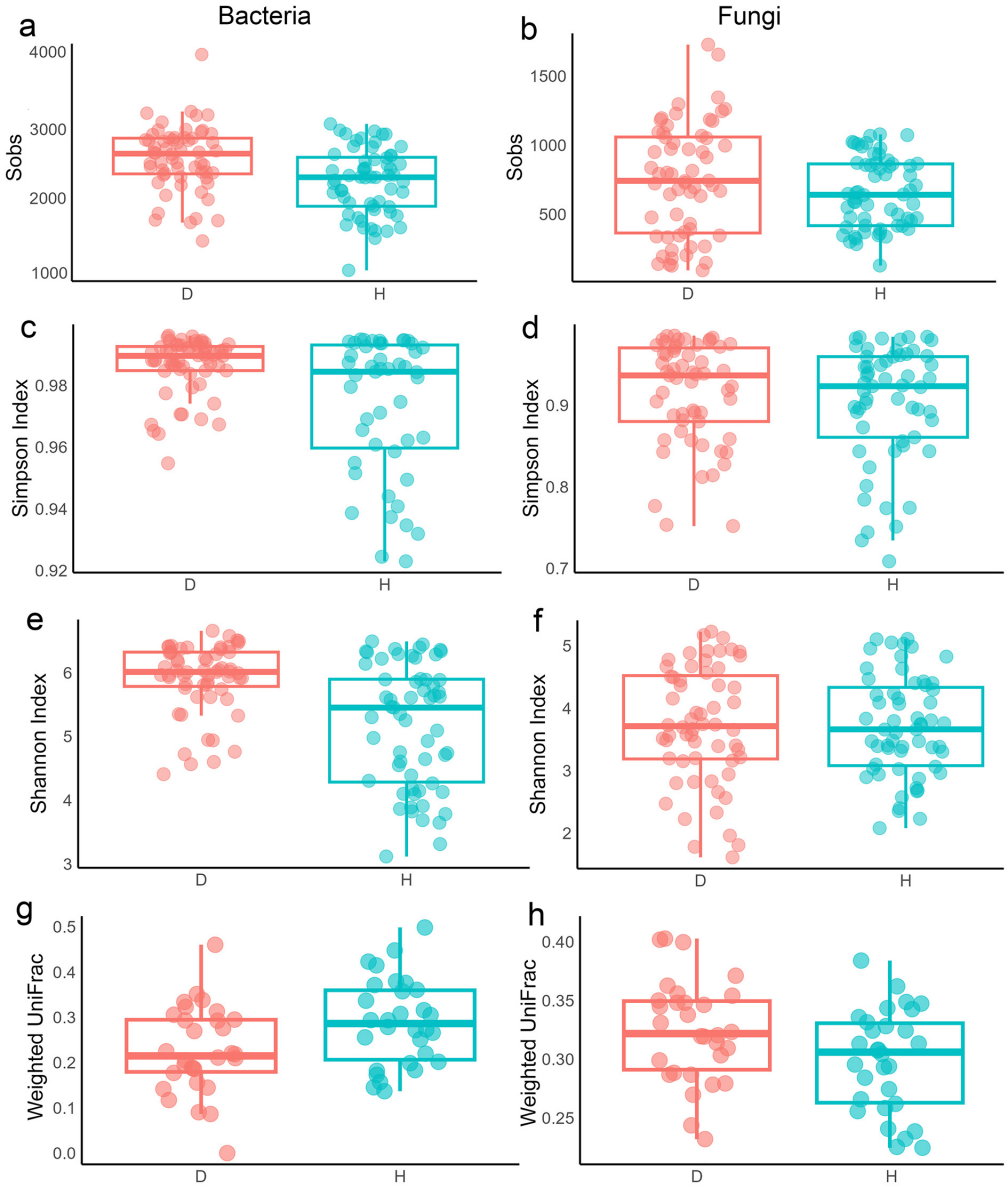
Alpha and beta diversity of microbial community responses to drought during dehydrated (D) and hydrated (H) states. **(a, b)** Sobs diversity index of bacterial **(a)** and fungal **(b)** communities. **(c, d)** Simpson diversity index of bacterial **(c)** and fungal **(d)** communities. **(e, f)** Shannon diversity index of bacterial **(e)** and fungal **(f)** communities. **(g, h)** Beta diversity of bacterial **(g)** and fungal **(h)** communities between hydrated and dehydrated groups based on the weighted UniFrac distance matrices.

The overall richness (Sobs, ACE, and Chao1 indices) of bacterial and fungal communities was slightly lower in the dehydrated (D) group than in the hydrated (H) group (*p* < 0.001; Figures 6a, b and Supplementary Figures S15–S17) when populations were pooled, consistent with single-population patterns except for fungal communities of GB and GS (*p* > 0.05–0.01; Supplementary Figures S15–S17). Species evenness and diversity followed the same trend (*p* < 0.001; Figures 6c–f), with similar results at the single-population level except for fungi in GB and QT (*p* > 0.05; others *p* < 0.01; Supplementary Figures S18, S19). Weighted UniFrac showed significant bacterial (*p* < 0.001) but not fungal differences between D and H overall (Figures 6g, h). At the single-population level, bacterial communities differed significantly in EJIA, GB, and GS populations (*p* < 0.01 to 0.001) but not in QT and WS (Supplementary Figure S20). For fungi, beta diversity was significant in GS and WS, with increased weighted beta diversity during drought. Beta diversity based on unweighted UniFrac distance showed that bacterial beta diversity in D and H groups differed significantly (*p* < 0.01), while fungal beta diversity was not significantly different when all populations were analyzed together (Supplementary Figure S21). At a single-population level, bacterial communities differed significantly between D and H groups (*p* < 0.05; Supplementary Figure S21) in the QT population (reduced during drought), whereas in other populations, differences were not significant. For fungal communities, unweighted beta diversity increased during drought (D vs. H). This increase was significant in EJIA and WS populations (*p* < 0.05 to 0.001) but not in other populations (Supplementary Figure S21). PCA explained 29.93%/10.03% of bacterial and 19.73%/12.68% of fungal variance, showing overlap when pooled (Supplementary Figure S22), but clearer D vs. H separation was observed at the single-population level (except bacterial QT), with PC1 variances of 20.14% (EJIA bacteria) and 20.4% (GB fungi).

**Figure 7:**
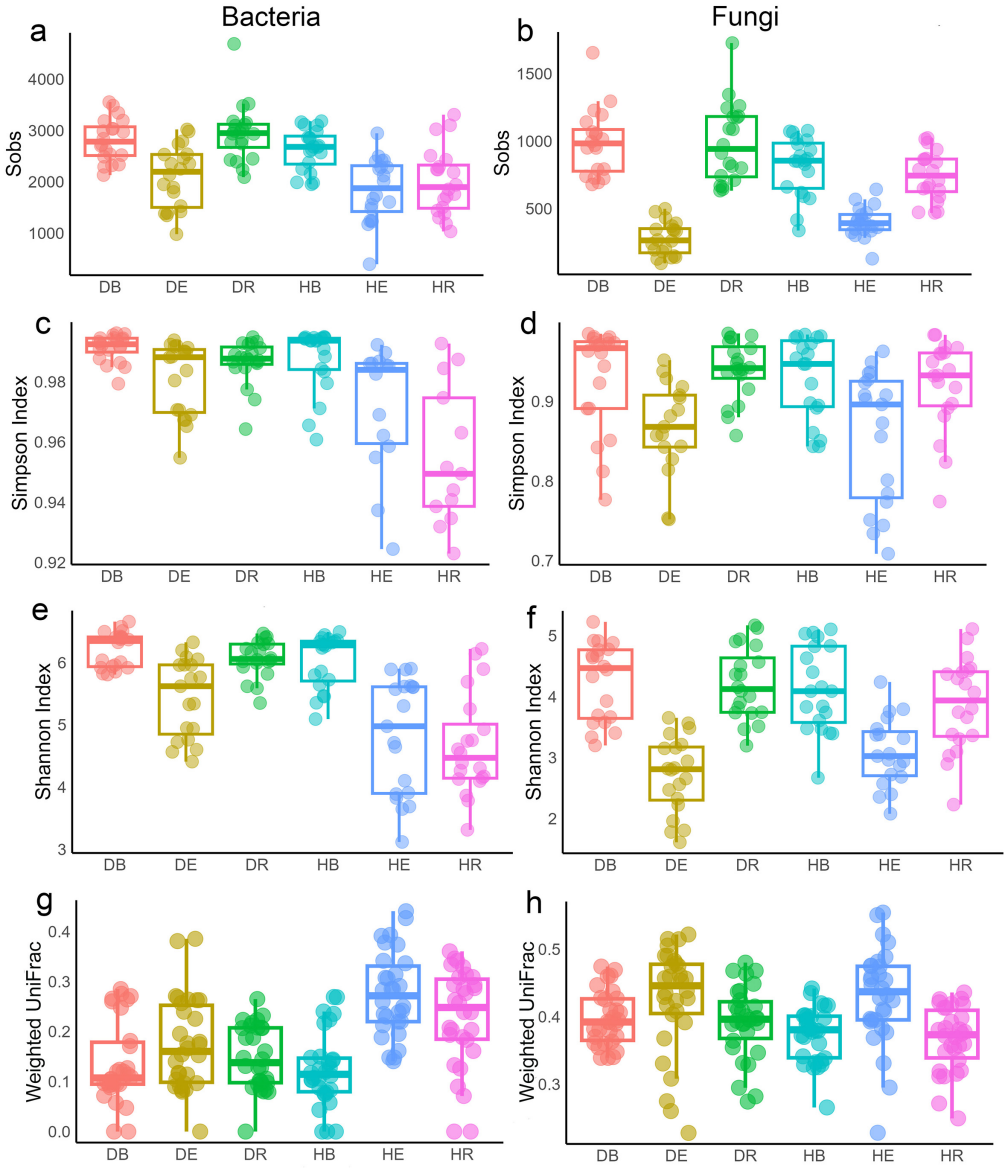
Alpha and beta diversity of microbial communities responses across six compartments during dehydrated and hydrated states. **(a, b)** Sobs diversity index of bacterial **(a)** and fungal **(b)** communities. **(c, d)** Simpson diversity index of bacterial **(c)** and fungal **(d)** communities. **(e, f)** Shannon diversity index of bacterial **(e)** and fungal **(f)** communities. **(g, h)** Beta diversity of bacterial **(g)** and fungal **(h)** communities between hydrated and dehydrated groups based on the weighted UniFrac distance matrices. DB, dehydrated bulk soil; DE, dehydrated root endosphere; DR, dehydrated rhizosphere; HB, hydrated bulk soil; HE, hydrated root endosphere; HR, hydrated rhizosphere.

During drought, alpha diversity differed by compartment. Bacterial richness increased in all compartments, significant only in DR vs. HR (*p* < 0.001), while fungal richness decreased in DE compared to HE but increased in DR compared to HR (*p* < 0.05 to 0.001; Figures 7a, b). This pattern was consistent with analyses of species richness at the single-population level for both bacterial and fungal communities (*p* < 0.05 HR vs. DR compartments of WS population and *p* > 0.05 in other populations; Supplementary Figures S23–S25). Overall species evenness increased in bacterial communities during drought (*p* < 0.05), but decreased in the fungal community in DE compared to HE (*p* > 0.05 Figures 7c, d) when all populations were analyzed together. This pattern was almost similar at the single-population level, except bacterial species evenness in DE decreased compared to HE in EJIA and WS populations (*p* > 0.05), and fungal species evenness in DE increased in WS population compared to HE (*p* > 0.05; Supplementary Figure S26). Overall species diversity (Shannon index) was also consistent with previous analyses (*p* < 0.05; Figures 7e, f) for both bacterial and fungal communities. At the single-population level, diversity of the bacterial community in the QT population DE compartment decreased compared to HE compartment (*p* > 0.05; Supplementary Figure S27), while fungal community diversity in the QT endosphere compartment increased in DE compared to HE, opposite to the patterns observed in other populations (*p* > 0.05). Beta diversity based on weighted UniFrac distance showed that all dehydrated compartments had decreased beta diversity compared to their hydrated counterparts for bacterial communities, while the opposite pattern was observed in fungal communities when all populations were analyzed together (*p* < 0.05 Figures 7g, h).

### Drought stress drives differential enrichment of microbial communities

LefSe analysis (LDA > 3, *p* < 0.01, FDR <0.05) revealed stronger microbial shifts under drought, with more differentially abundant bacterial taxa across compartments and populations than under hydration (Supplementary Tables S8, S9). This pattern varied by population, with EJIA and WS showing the largest changes, whereas fungal communities remained comparatively stable, displaying only modest shifts. Differential abundance of bacterial and fungal communities across three compartments of D and H groups revealed several significantly differentially abundant top 10 phyla and genera taxa (Supplementary Figure S28; *p* > 0.05 to <0.001). Among bacterial phyla, Proteobacteria, Actinobacteriota, Acidobacteriota, Myxococcota, Firmicutes, Bacteroidota (except EJIA population), and Chloroflexi were universal across populations, as observed previously. In the EJIA population, the most differentially abundant genera in the H group were *Burkholderia-Caballeronia-Paraburkholderia, Lactococcus* (highly abundant in HE and HR compartments), and *Bacillus*, whereas *Bradyrhizobium*, norank Acidobacteriales, norank Xanthobacteraceae, *Acidothermus*, and *Granulicella* were enriched in the D group compartments (*p* < 0.001). In the GB population, bacterial genera such as *Bacillus, Pseudomonas, Burkholderia-Caballeronia-Paraburkholderia, Allorhizobium-Neorhizobium-Pararhizobium-Rhizobium*, and *Mycobacterium* were more abundant in H group compartments, while unclassified Enterobacteriaceae, norank_f__67-14 and *Microlunatus* were abundant in D group (*p* < 0.05 to 0.001). In the GS population, patterns were similar to GB, except *Pseudonocardia* and *Microlunatus* were enriched in D group samples (*p* < 0.01 to 0.001). In the QT population, *Burkholderia-Caballeronia-Paraburkholderia* was abundant in DR, HR, and HE compartments, whereas *Allorhizobium-Neorhizobium-Pararhizobium-Rhizobium* (abundant in DE), norank_f__67-14, *Mycobacterium*, and *Pseudonocardia* were differentially abundant across all compartments of D and H groups (*p* < 0.05 to 0.001). In the WS population, unclassified Enterobacteriaceae, *Allorhizobium-Neorhizobium-Pararhizobium-Rhizobium, Bacillus, Pseudomonas* and *Paenarthrobacter* were mainly enriched in HR and HE compartments of H group, whereas norank_f__67-14, *Microlunatus, Pseudonocardia*, and norank Xanthobacteraceae were enriched in all compartments of both H and D groups (*p* < 0.05 to 0.001).

For fungal communities, core phyla were dominant and universal across all populations. However, genus-level taxa were varied between populations. For example, in EJIA, unclassified Hyaloscyphaceae (abundant in DE compartment), *Trechispora* (abundant in D group compartments), *Mortierella* (abundant in H group compartments), unclassified Fungi, unclassified Capnodiales (abundant in DE compartment), unclassified Helotiales, *Penicillium*, and *Tomnetella* (abundant in H group compartments) were differentially abundant (*p* < 0.001). In GB, unclassified Fungi, *Penicillium, Mortierella, Tomnetella, Fusarium*, and *Trichoderma* were differentially abundant (*p* < 0.01 to 0.001). In GS, *Sebacina* (abundant in D group compartments), unclassified Fungi, *Penicillium*, unclassified Herpotrichiellaceae (abundant in DE compartment), unclassified Ascomycota, unclassified Pannariaceae, *Tomnetella*, and *Cyphellophora* were differentially abundant (*p* < 0.05 to 0.001). In QT, unclassified Thelephoraceae (abundant in D group compartments), unclassified Fungi, *Sebacina*, unclassified Herpotrichiellaceae, unclassified Pleosporales, and unclassified Eurotiomycetes were differentially abundant (*p* < 0.001). In WS, unclassified Pleosporales and unclassified Herpotrichiellaceae were significantly abundant in H group compartments, whereas unclassified Fungi, unclassified Ascomycota, *Hygrocybe*, unclassified *Helotiales*, unclassified Agaricales, and *Cyphellophora* were significantly abundant in D group compartments (with the last three enriched in DE).

### Changes in microbial community function across compartments and in response to drought

To simplify interpretation, populations were pooled, as major functional trends were largely conserved across populations. This approach enabled clearer assessment of microbial responses to compartment and drought variation.

Given the potential role of microbial communities in enhancing plant drought resilience, we examined functional differences across plant compartments and between drought-stressed (D) and well-watered (H) *O. mileensis* samples. From pooled data, 23 Clusters of Orthologous Groups (COG) functional categories were identified across the bulk soil (B), rhizosphere (R), and endosphere (E) (Supplementary Figure S29). Bulk soil was enriched in functions linked to protein synthesis, DNA repair, protein turnover, nucleotide and lipid metabolism, defense, coenzyme metabolism, and chromatin structure. Rhizosphere was enriched in cell cycle control and chromosome partitioning, whereas the endosphere showed enrichment in inorganic ion transport, chromatin structure, and functions of unknown category. Comparisons revealed 18 significant differences between B and R, 19 between B and E, and only 9 between R and E (*p* < 0.05; FDR <0.05).

Drought induced broad functional shifts (Supplementary Figures S30a–c). KEGG analysis showed enrichment in signaling, substance dependence, infectious disease, excretory system, and cell growth/death pathways, while the sensory system and environmental adaptation increased but lost statistical significance after FDR correction. At the COG level, 13 categories were enriched under drought, including ribosomal function, signal transduction, secondary metabolism, DNA repair, lipid metabolism, trafficking, energy production, defense, cytoskeleton, coenzyme metabolism, chromatin structure, cell wall biogenesis, and cell cycle (*p* < 0.05; FDR <0.05). Hydrated plants showed higher transcription, inorganic ion transport, cell motility, carbohydrate metabolism, and amino acid metabolism. MetaCyc analysis supported these trends, with drought enhancing nucleotide, nitrogen, energy, and amino acid metabolism, while hydration favored stress response, degradation, and carbohydrate/aromatic metabolism.

Fungal functional roles also shifted (Supplementary Figure S31). Of 27 annotated functions, 9 (e.g., algal parasite, endophyte, ericoid mycorrhizal) were enriched under hydration, whereas 5 (e.g., ectomycorrhizal, endomycorrhizal, dung saprotroph, lichenized) increased under drought (*p* < 0.05; FDR <0.05). Compartment-specific patterns included ericoid mycorrhizal enrichment in B, endomycorrhizal in R, and arbuscular/orchid mycorrhizal and wood saprotrophs in E. Hydration further modulated these patterns, with parasites enriched in HB/HR, while drought favored dung saprotrophs and ectomycorrhizal fungi in DB/DR and lichenized fungi in DE.

## Discussion

Plants actively shape and respond to their associated microbiomes through diverse mechanisms, such as phytohormonal signaling and defense responses, creating a dynamic network of interactions that influence microbial diversity, soil community structure, and ecosystem feedbacks (Jacoby et al., 2017; Angulo et al., 2022; Chialva et al., 2022). These interactions ultimately offer opportunities to enhance plant nutrition and support sustainable agriculture through microbiome engineering. Although some studies on resurrection plants have identified plant growth-promoting microbes, particularly in the rhizosphere and endosphere, these investigations have largely been limited to single or nearby sampling locations, thereby potentially underrepresenting the microbial diversity associated with a given plant species (Lozo et al., 2023; Sun et al., 2024; Tebele et al., 2024, 2025). In this study, we collected root samples from five distinct populations of *O. mileensis* and performed sequencing of both bacterial and fungal communities to gain new insights into microbial diversity and to deepen our understanding of microorganisms associated with desiccation. We observed that microorganisms varied across compartments. In contrast to the strong influence of compartment-specific niches, extreme dehydration stress had a relatively weaker impact on the diversity, composition, and functional traits of microbial communities. This suggests that *O. mileensis* plants maintain a stable, well-structured microbiome across different compartments under natural conditions.

### Conserved core phyla, genera, and population-specific taxa shape the microbial community of *O. mileensis*

We determined that microbial taxa at higher taxonomic rank (phylum) are conserved across populations but differ relatively at lower taxonomic ranks (e.g., genus). Previous research has shown that Proteobacteria, Actinobacteriota, Firmicutes, Acidobacteriota, and Bacteroidota phyla were primarily dominant in the bulk soil, rhizosphere, and endosphere of several resurrection plants from Africa, Eurasia, Brazil, and East Asia, despite the substantial geographic distance between those plants (Lozo et al., 2023; Sun et al., 2024; Tebele et al., 2024; Pinto et al., 2025). Consistently, we identified these as core taxa, along with secondary core phyla such as Myxococcota and Chloroflexi, which were present across all populations, and Nitrospirota, which was specifically enriched in the WS and QT populations. These results align with previous observations in other plant systems, where Myxococcota showed drought responses in crops (Guo et al., 2023) and *Angelica sinensis* (He et al., 2025), Chloroflexi increased under drought in rice (Santos-Medellín et al., 2017), and Nitrospirota appeared associated with wild oat roots (DeAngelis et al., 2009) and tropical soils (Yeoh et al., 2017). Similarly, whether populations are analyzed together or separately, they had almost the same core phyla of fungi, including Ascomycota, Basidiomycota, and Mortierellomycota (unusually high in hydrated rhizosphere and root endosphere of GS population samples). These taxa were also found in previous studies (Orgiazzi et al., 2013; Abdelrazek et al., 2020; Sun et al., 2024; Tebele et al., 2024) as predominant members of soil–root compartments, suggesting that geographical distance does not strongly affect these taxa. Despite the stability of phylum-level taxa, we observed that soil–root compartments have relatively different genera and abundance in *O. mileensis*. When all populations were analyzed together, the core genera included *Burkholderia-Caballeronia-Paraburkholderia*, mostly associated with the hydrated state of all three compartments. This group contains a large number of species widely distributed across humans, animals, and plants (Estrada-de Los Santos et al., 2013; Depoorter et al., 2016). Another core taxon, *Bacillus*, associated with rhizosphere and root endosphere, is also mainly found in many crops and habitats (Germida et al., 1998; Andrić et al., 2020). The next core taxa, *Allorhizobium-Neorhizobium-Pararhizobium-Rhizobium* and *Pseudomonas*, represent large groups of closely related species mostly associated with legumes and other plants (Dresler-Nurmi et al., 2009). Previous studies conducted across tropical regions have also shown that core root-associated microorganisms such as *Bradyrhizobium, Mycobacterium*, and *Microlunatus* were evolutionarily conserved across a wide range of plant phyla (Yeoh et al., 2017). However, their abundance levels differed across populations (see Supplementary Figure S4). In addition, we identified some population-specific genera among the top 10 most abundant bacteria. For example, *Lactococcus* was abundant in the hydrated rhizosphere/root endosphere, while *Granulicella* was abundant across all compartments of the EJIA population. *Microbacterium* was unique and abundant in the root endosphere of the GS population; *Mesorhizobium*, in all compartments of the QT population; *Rubrobacter*, in the bulk soil and rhizosphere; and *Paenathrobacter* (a core taxon) in the rhizosphere of the WS population. Finally, *Pseudonocardia* was found in almost all compartments of the GS, QT, and WS populations (as a core taxon). In fungal community, when all populations were analyzed, *Mortierella* and *Paraboeriemia* were identified as core taxa, both previously recognized as central metacommunity hubs in Japanese forests (Toju et al., 2018) and bananas (Birt et al., 2023). The next core taxa, unclassified Pleosporales and Herpotrichiellaceae, were found in cycad coralloid roots and other many plant roots (Glynou et al., 2018; Wang et al., 2023). The remaining taxa, *Penicillium* (core taxa) and *Sebacina* (abundant in GS and QT populations but less abundant in other populations), have been reported in diverse plant roots, including those of *Argania spinosa*, wheat, and sunflower (Weiß et al., 2011; Urooj et al., 2018; Abdallah et al., 2019; Schlatter et al., 2020), *Saitoszyma* (not a core taxon), unclassified Ascomycota (core taxon), and Agaricales (not a core taxon) have also been documented in the forest plants (Toju et al., 2013; Wang et al., 2023).

### Drought selects for stress tolerance and homogenizes bacterial communities while differentiating fungal assemblages in *O. mileensis*

Our alpha diversity showed a consistent decline from the bulk soil to the root endosphere in both bacterial and fungal communities, regardless of hydration status. This gradient was accompanied by a decrease in beta diversity from the endosphere and rhizosphere toward the bulk soil, indicating greater compositional similarity with increasing distance from the roots. These patterns align with previous studies, which highlight bulk soil as a reservoir of higher richness but lower differentiation, while root-associated compartments reflect strong plant-driven ecological filtering (Wang et al., 2016; Wang C. et al., 2020; Xiong et al., 2021; Tebele et al., 2024).

Under drought, alpha diversity increased whereas beta diversity decreased across all compartments, consistent with studies on rice and wheat (Wu et al., 2024), whereas a recent study of Velloziaceae species showed no significant shift in drought and non-drought samples (Pinto et al., 2025). This pattern reflects stronger deterministic processes, where filtering and host selection favor drought-adapted generalists such as Actinobacteriota (Wu et al., 2024). Bacterial alpha diversity increased significantly in D samples compared to H samples at both combined and individual population levels, while weighted beta diversity declined (non-significant in two populations). Unweighted beta diversity showed only minor, non-significant increases in the GS and WS populations. These results suggest drought homogenizes bacterial communities by selecting for stress-tolerant taxa and reducing niche differentiation in *O. mileensis* (Fierer et al., 2003; Wang P. et al., 2020).

Fungi showed similar increases in alpha diversity under drought but a contrasting beta diversity response: both weighted and unweighted beta diversity increased in most cases (significantly in two populations), driven by shifts in dominant and rare taxa. This suggests that host plants exert particularly strong selective pressures on rhizosphere fungal communities during drought, potentially shaping microbial assemblages that enhance host fitness (Pan et al., 2024).

### Hydration-driven assembly of microbial communities reveals potential drought-responsive bacterial and fungal taxa across compartments

Proteobacteria emerged as the dominant phylum across all compartments, demonstrating its central role in plant growth promotion, nutrient cycling, and stress resistance (Liu et al., 2017; Zhang et al., 2022). Its abundance was significantly higher in the endosphere and rhizosphere than in the bulk soil, unlike that of Actinobacteriota, though dominant genera varied among *O. mileensis* populations. The Gammaproteobacteria group *Burkholderia-Caballeronia-Paraburkholderia*, significantly enriched under hydration conditions in EJIA, GB, and QT populations, supports plant fitness through nitrogen-dependent and -independent mechanisms and confers stress tolerance (Madhaiyan et al., 2021; Pal et al., 2022). Similarly, *Allorhizobium-Neorhizobium-Pararhizobium-Rhizobium* (ANPR) strains, more abundant in hydrated endospheres in three populations but in dehydrated endospheres in two, are known to enhance plant drought and salinity tolerance by accumulating osmoprotectants such as trehalose, activating host antioxidant systems, and improving nitrogen uptake (Zahran, 1999; Reina-Bueno et al., 2012; Datta et al., 2015).

Acidobacteriaceae showed compartment-specific patterns consistent with plant growth-promoting functions (Reis and Teixeira, 2015; Pedraza, 2016). In metal-rich karst soils (Qu and Han, 2022), *Granulicella* increased under drought in EJIA, potentially contributing to metal ion homeostasis and stress adaptation (Costa et al., 2020). *Acidothermus*, also enriched in dehydrated soils of EJIA, has been linked to organic matter decomposition and nutrient uptake under heat stress (Lin et al., 2022).

*Bacillus* spp., enriched in hydrated states across compartments and in dehydrated rhizospheres (EJIA, GB, WS), are established plant growth-promoting bacteria (PGPB) that enhance water and nutrient uptake, phytohormone synthesis, and drought tolerance (Radhakrishnan et al., 2017; Lahlali et al., 2022; Yousfi et al., 2024). *Bradyrhizobium* also increased during dehydration, particularly in the endospheres of EJIA, GB, GS, and QT, paralleling reports in peanuts where inoculation enhanced drought tolerance through increased root biomass, nodule number, and stress-related gene expression (Barbosa et al., 2018; Brito et al., 2019), while also improving physiological responses and antioxidant enzyme activities—crucial adaptations for resurrection plants such as *O. mileensis* (Sherwin and Farrant, 1998; Li et al., 2014).

Other drought-responsive taxa included *Microlunatus*, enriched in the rhizospheres and endospheres of GB, GS, and WS populations, which presents an interesting case. Known for polyphosphate accumulation (Nakamura et al., 1995), it may release phosphate under drought via root exudate stimulation, supporting host nutrition. We hypothesize that drought-induced root exudates (e.g., organic acids) may stimulate *Microlunatus* to release stored phosphate, enhancing plant availability during water scarcity. Although EPS/trehalose production has not been confirmed in *Microlunatus*, related Actinobacteria and polyphosphate-accumulating bacteria (PAOs) are known to synthesize these drought-mitigating compounds (Kour et al., 2019).

*Pseudomonas mosselii* and *P. citronellolis* were highly abundant in hydrated rhizosphere/endosphere samples of all populations. These promote plant growth by enhancing the uptake of N, P, Mg, S, and Fe (Sultana et al., 2024) and help sustain chickpea under metal stress by maintaining cellular homeostasis and normalizing antioxidative/carbohydrate enzymes (Adhikary et al., 2019, 2022). We hypothesize that they are critical for *O. mileensis*' post-drought recovery.

*Pseudonocardia*, which exhibited strong root colonization during drought (GS, QT, and WS populations), is associated with IAA and antimicrobial production (Riahi et al., 2022; Tedsree et al., 2022), likely aiding survival. While its drought enrichment mechanism remains unclear, it may contribute to *O. mileensis* survival during water scarcity. Similarly, *Microbacterium*, consistently detected in GS endospheres under both hydration states, likely contributes through auxin production, ACC deaminase (reducing ethylene stress), phosphate solubilization (Zhang et al., 2017), and pathogen protection (Barnett et al., 2006; Pereira et al., 2007; Madhaiyan et al., 2010; Alves et al., 2014). *Rubrobacter*, abundant in WS and present in other populations, mirrors observations in drought-stressed *Vigna subterranea*, where its enrichment was proposed to aid plant survival (Ajilogba et al., 2022). Its stress-responsive enzymes (e.g., SOD) and potential RubisCO-mediated C-fixation suggest roles in ROS scavenging and metabolic maintenance during water deficit, consistent with other drought-resilient Actinobacteria (Starke et al., 2017). *Paenarthrobacter*, abundant in hydrated rhizospheres (particularly in WS) and prevalent in other populations, may function similar to *P. nitroguajacolicus* strain P1, improving nutrient availability and photosynthesis (Salimi et al., 2023), potentially aiding *O. mileensis*' post-rewatering recovery.

Fungal communities play indispensable roles in plant growth and stress tolerance through diverse symbiotic relationships. Ascomycota dominated across populations, while Basidiomycota increased under drought. The endophytic genus *Paraboeremia* was particularly prevalent, with *P. litseae* producing antimicrobial compounds that protect hosts (Ming et al., 2022). An unclassified Hyaloscyphaceae species reached high abundance in droughted EJIA endospheres, similar to *Lachnum* spp., which promote drought tolerance via SOD enhancement and root growth (Lou et al., 2022).

Among Basidiomycota, *Saitozyma podzolica* dominated hydrated rhizospheres (particularly in QT and EJIA), with reported biocontrol activity against Fusarium wilt (Das et al., 2023). Another intriguing finding was the drought-enriched *Trechispora* sp. in EJIA endospheres. *Trechispora* sp., enriched in EJIA droughted endospheres, may play novel endophytic roles despite its ectomycorrhizal associations (Dunham et al., 2007; Vanegas-León et al., 2019).

Hydration-specific fungal signatures included *Mortierella alpina*, abundant in hydrated samples across populations, contributing antimicrobial and protease activities (Sakuradani et al., 2009; Gawas-Sakhalkar and Singh, 2011). Specialized distributions were also noted for *Podila clonocystis* in bulk soil/rhizosphere and unclassified Helotiales in EJIA (in both hydration states) and WS dehydrated endosphere, consistent with known nutritional symbioses (Grelet et al., 2009; Tedersoo and Smith, 2013; Hill et al., 2019). The diverse *Penicillium community* (>25 species across populations) spanned beneficial taxa such as *P. virgatum* and *P. kongii*, which exhibit antibacterial properties (Liu et al., 2018; Kwon et al., 2021; Li et al., 2024), alongside contaminants (Visagie et al., 2014; Houbraken et al., 2020). Drought-enriched *Tomentella species* (*T. papuae, T. incrustata*, etc.) with known ectomycorrhizal associations (Jakucs et al., 2015; Nouhra et al., 2015) may enhance *O. mileensis* survival through nutrient exchange under water stress.

Several rare taxa warrant attention. For instance, *Alfoldia* sp. (Wijayawardene et al., 2024) consistently appeared in QT, WS, and GB endospheres, though its functions remain unknown. Similarly, unclassified Agaricales showed drought enrichment in WS endosphere and GB compartments, presenting research opportunities given this order's ecological diversity (Dwivedi et al., 2017). Particularly compelling was *Cyphellophora* sp., which demonstrated significant drought enrichment in GS and WS endosphere/rhizosphere. Its documented IAA production (Bi et al., 2024) and association with sugarcane smut resistance (Duan et al., 2023) suggest potential mechanisms for enhancing *O. mileensis* drought tolerance, possibly through phytohormone-mediated stress responses similar to those observed in IAA-treated *Arabidopsis* (Almazroue, 2014).

Our study reveals distinct shifts in microbial functional potential across root-associated compartments and in response to drought in *O. mileensis*, underscoring the dynamic interplay between plant microhabitats, environmental stress, and microbial community function. By aggregating samples from five populations, we focused on identifying broadly conserved trends rather than population-specific variability, an approach justified by the consistency in functional patterns across populations and the need for analytical clarity.

The functional analyses of bacteria revealed compartmental specialization. Bulk soil (B) was enriched in core metabolic processes such as translation, replication/repair, lipid metabolism, and defense functions, reflecting diverse, metabolically active communities. These functions are commonly associated with high microbial turnover and broad resource utilization capabilities typical of bulk soil environments (Fierer, 2017). In contrast, the rhizosphere was enriched in cell cycle/division, consistent with root exudate-driven microbial proliferation (Berendsen et al., 2012). Endospheres (E) harbored functions such as inorganic ion transport and uncharacterized pathways, indicating specialized nutrient exchange and novel symbioses. Chromatin-related functions enriched in bulk and endosphere suggest genomic adaptation to gradients and host filtering.

Drought had a marked influence on microbial functional profiles (Kozjek et al., 2022). KEGG and COG analyses revealed enrichment in stress-related pathways, including signal transduction, secondary metabolism, defense, and substance dependence, suggesting microbial investment in survival and plasticity (Xu and Coleman-Derr, 2019; Veach et al., 2020). Translation-related functions remained enriched under drought, possibly reflecting accelerated microbial activity due to increased competition or stress signaling. Conversely, hydrated states were enriched in transcription, ion transport, and carbohydrate metabolism, consistent with resource-rich environments (Lucci, 2019). MetaCyc supported these trends, showing drought-associated nitrogen, nucleotide, and energy metabolism, while hydration favored carbohydrate degradation and stress-response pathways (Lucci, 2019).

Fungal community profiling further revealed hydration-dependent ecological roles. Hydrated plants supported more diverse and saprotrophic (algal, bryophyte, leaf decomposers) fungal communities, whereas drought promoted symbiotic and stress-tolerant fungi, including ecto- and endomycorrhizal taxa, consistent with their known roles in water acquisition and stress mitigation (Poudel et al., 2021).

Compartment-specific fungal enrichment patterns also highlighted niche differentiation. For instance, endomycorrhizal fungi were more abundant in the rhizosphere, while arbuscular and orchid mycorrhizal fungi were enriched in the endosphere, suggesting selective recruitment by the plant under different hydration regimes. The enrichment of lichenized fungi under drought in the endosphere is possibly related to the plant's reduced photosynthesis in the dehydrated state.

Overall, our results support the hypothesis that both abiotic stress and plant compartmentalization are key drivers of microbial functional structure. The observed functional redundancy across populations and the coherence of trends across compartments and conditions justify our population-aggregated approach. Drought shifts microbial functions toward stress adaptation and symbiosis, reducing redundancy but potentially enhancing host resilience.

### Conservation implications for *O. mileensis*

As a PSESP, *O. mileensis* requires urgent conservation measures. Our research provides essential data to support its protection in three key ways. First, we identified potential drought-resistant and growth-promoting symbiotic microorganisms that enhance the plant's survival. These beneficial microbes could be used to boost the resilience of cultivated specimens for reintroduction programs. Second, we documented stable core microbial communities across populations, suggesting that soil microbiome conservation should be prioritized alongside plant protection in native habitats. Third, our functional analysis revealed microbial pathways that support stress tolerance, providing biomarkers to monitor plant health in changing environments. These findings directly inform conservation strategies by identifying critical microbial components that maintain *O. mileensis* populations. We recommend incorporating microbiome assessments into existing protection plans and using microbial augmentation to strengthen *ex situ* conservation efforts. This microbial-focused approach offers new tools to safeguard this endangered species against climate change and habitat degradation.

## Data Availability

The datasets presented in this study can be found in online repositories. The raw bacterial and fungal sequences presented in the study are deposited in the ENA database under PRJEB100941 and PRJEB100720 Project Accession numbers, respectively.
