# Supplementary material for: Comparative root associated microbial community analysis of Oreocharis mileensis, a resurrection plant species with extremely small populations
Source: Front Microbiol. 2025 Nov 4;16:1692695. doi: 10.3389/fmicb.2025.1692695 (PMC12623358; doi:10.3389/fmicb.2025.1692695)
Supplement: Supplementary file 1 [file Data_Sheet_1.pdf]

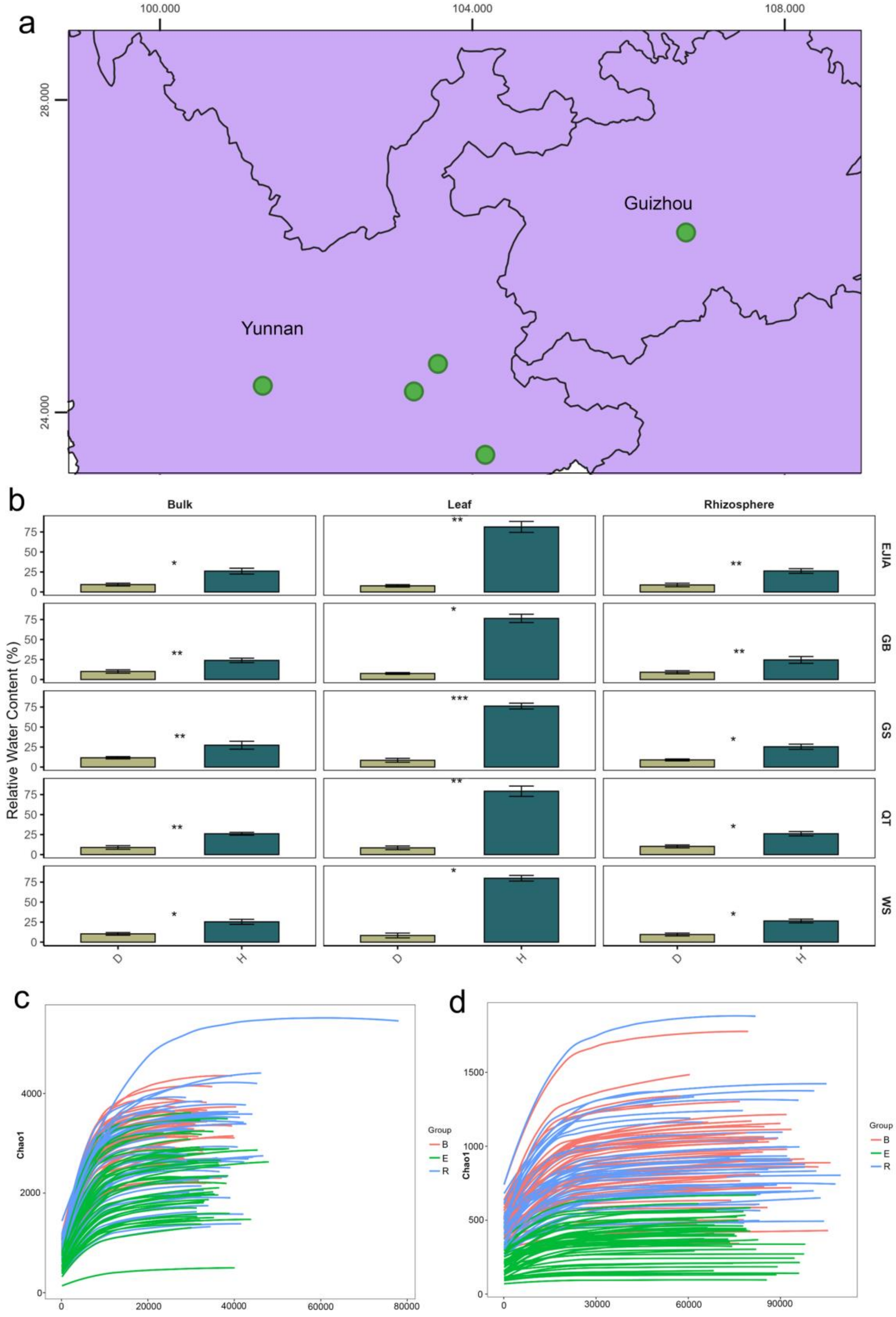

Figure S1 (a) sampling locations and (b) RWC difference between hydrated (H) and dehydrated (D) samples within each population. Rarefaction curves assessing microbial community sampling completeness of (c) bacteria and (d) fungi.

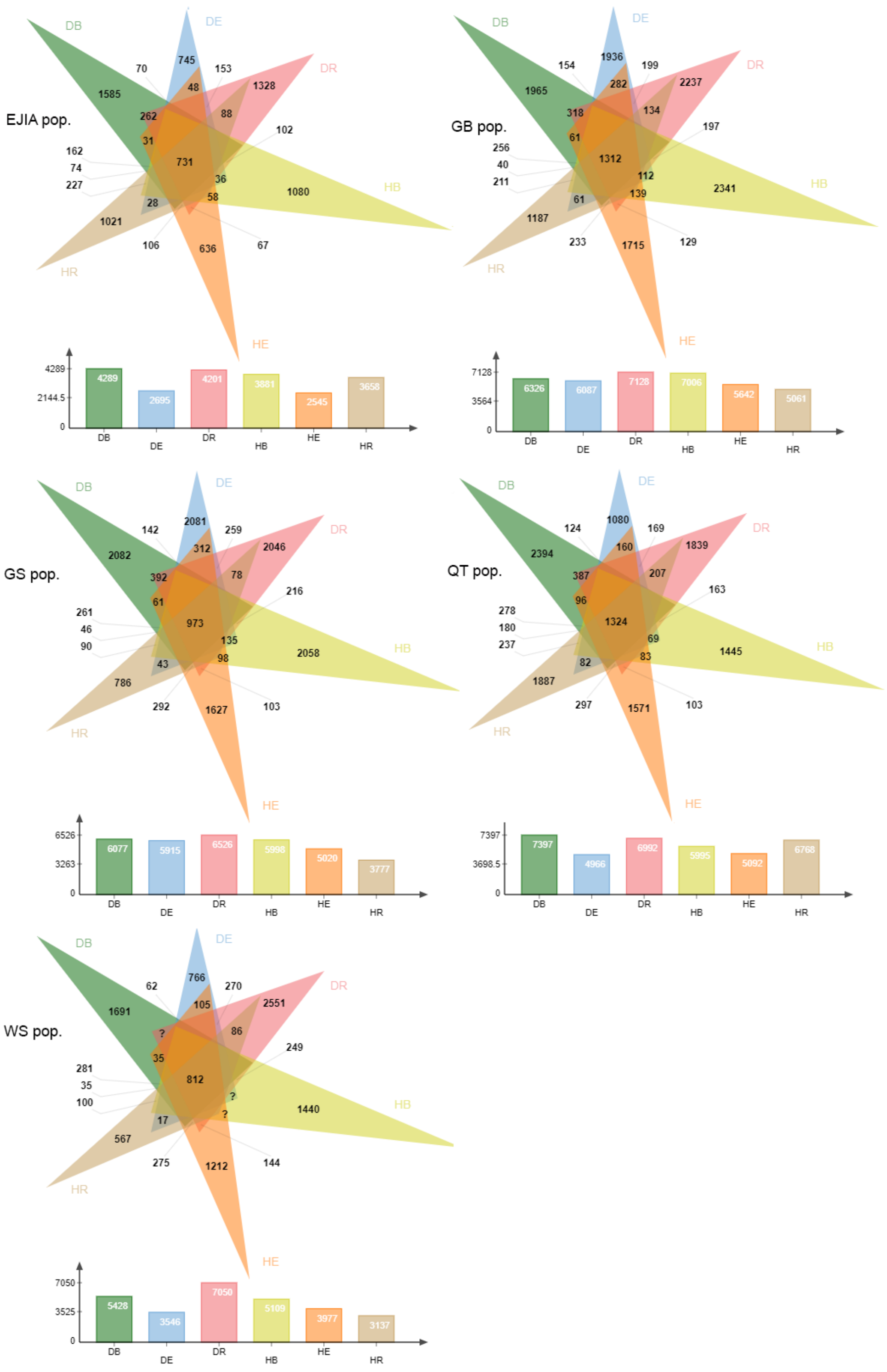

Figure S2 Comparison of operational taxonomic units in the compartments across hydrated and dehydrated states of bacterial composition within populations.

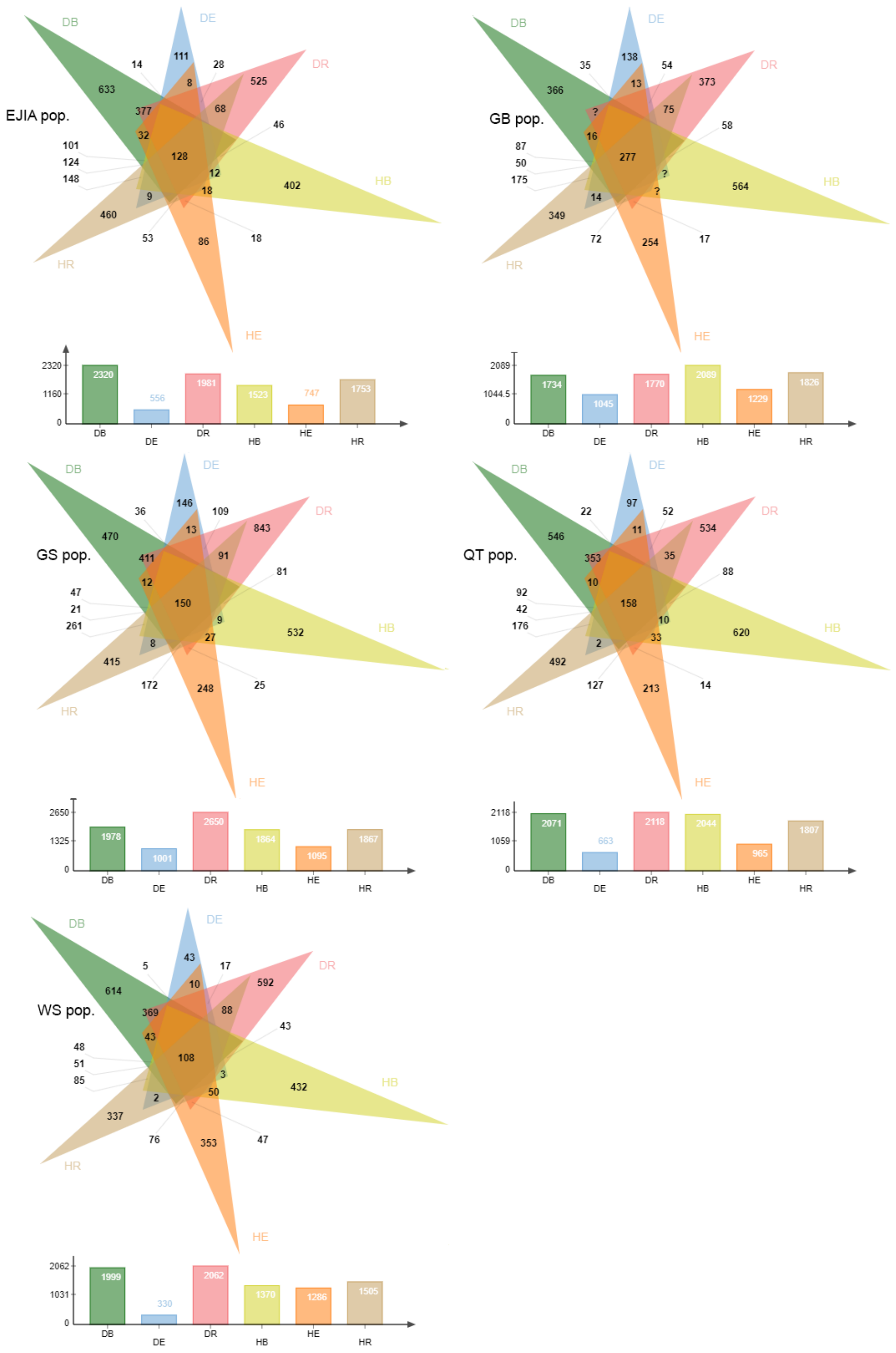

Figure S3 Comparison of operational taxonomic units in the compartments across hydrated and dehydrated states of fungal composition within populations.

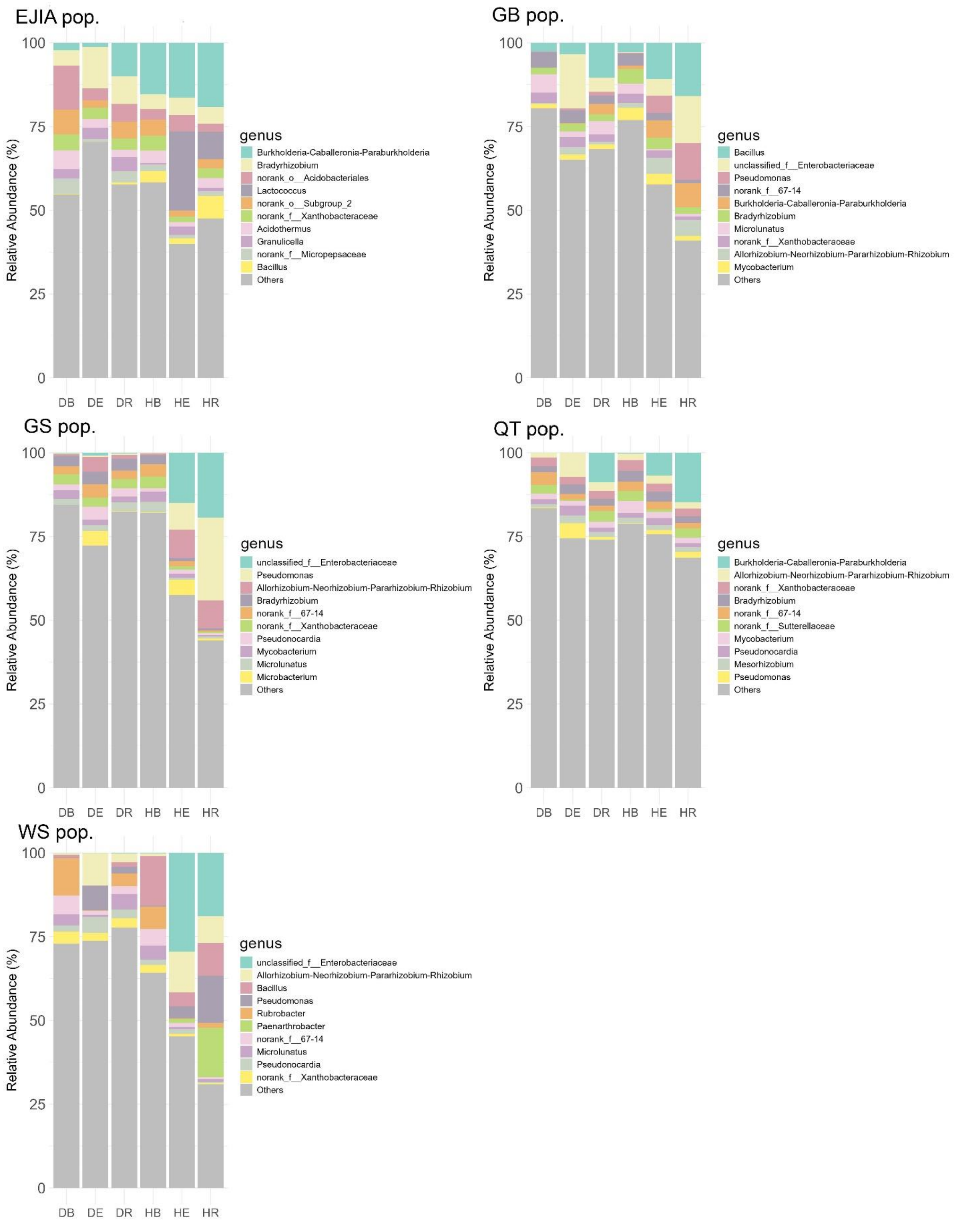

Figure S4 Relative abundance of bacterial genera within populations.

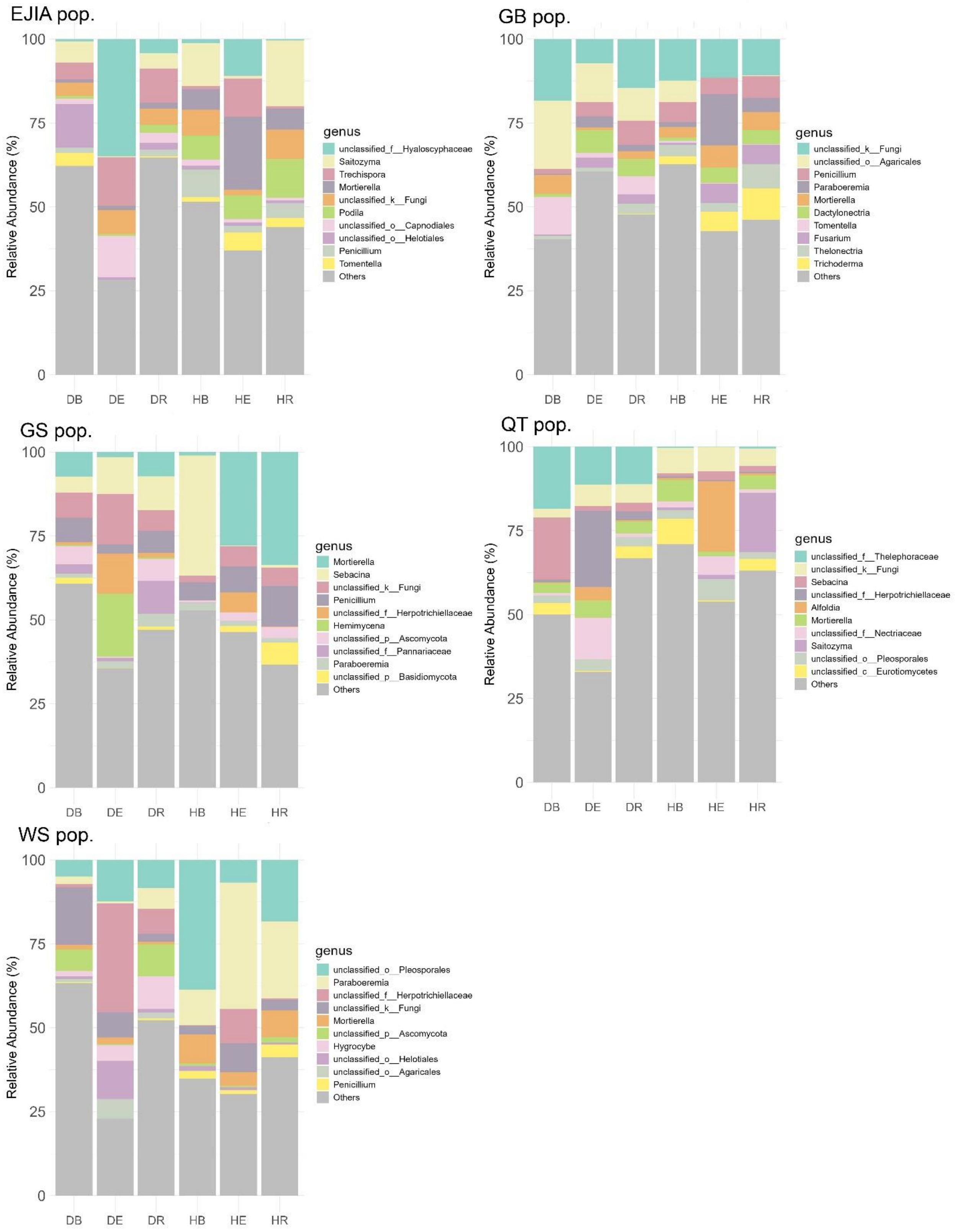

Figure S5 Relative abundance of fungal genera within populations.

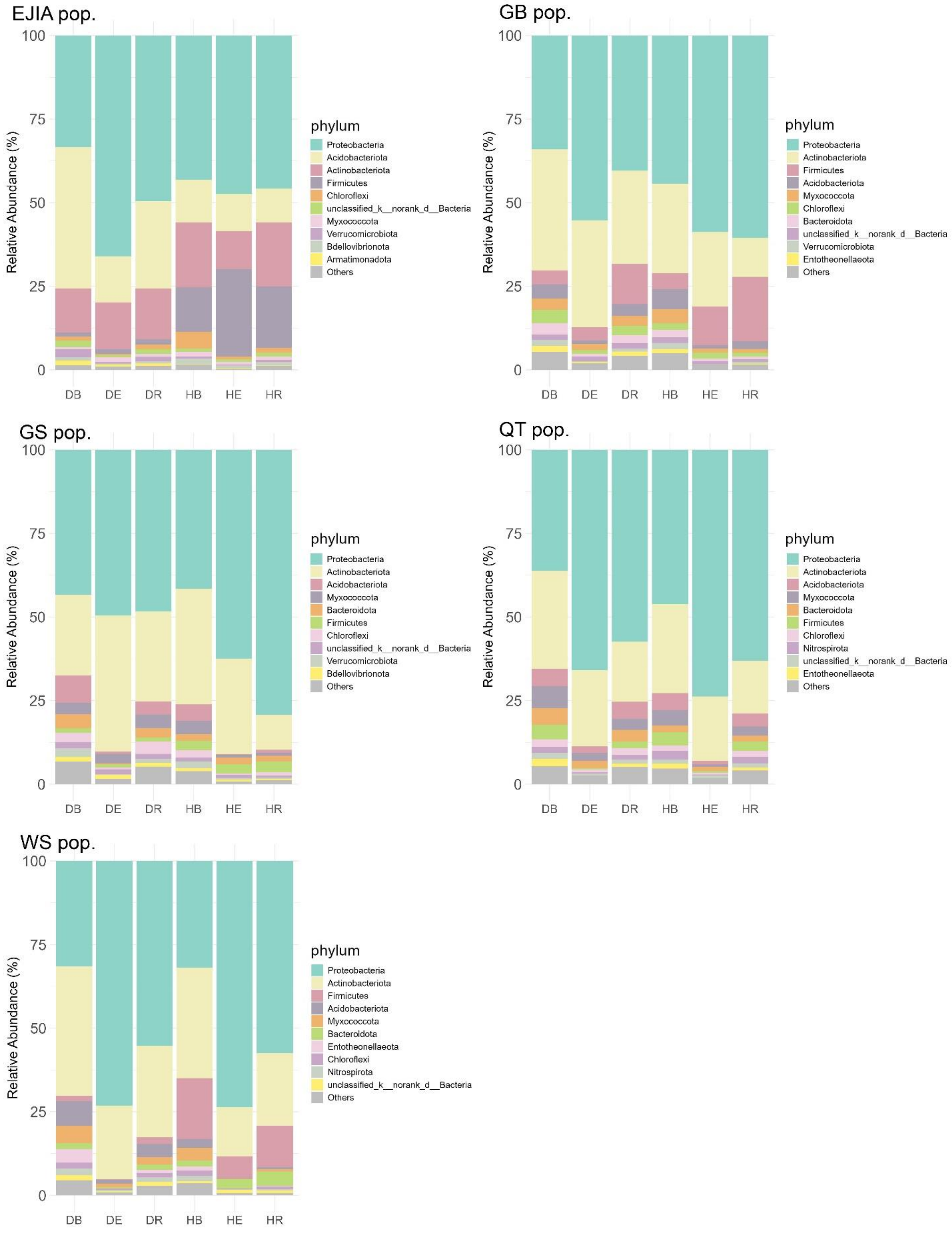

Figure S6 Relative abundance of bacterial phyla within populations.

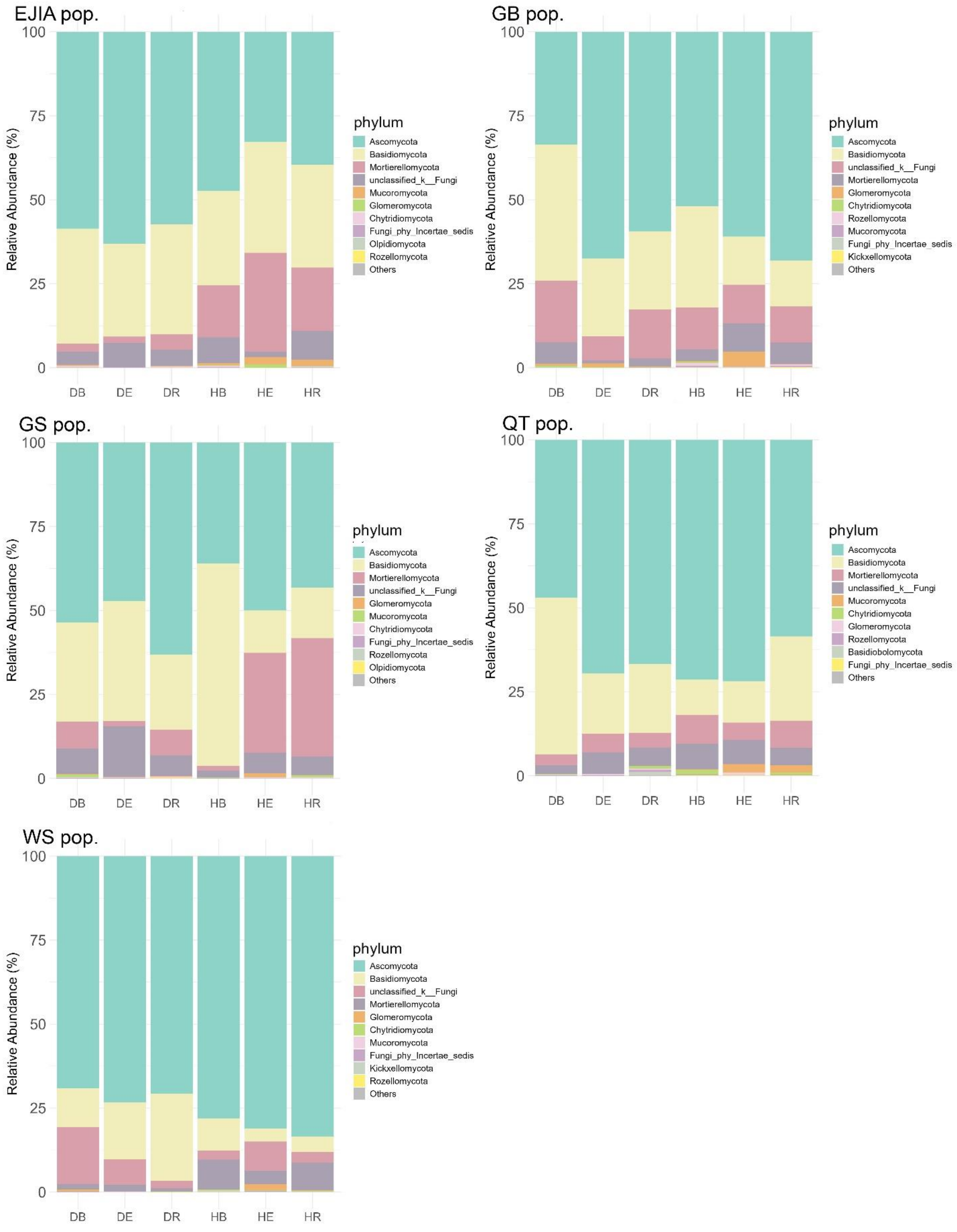

Figure S7 Relative abundance of fungal phyla within populations.

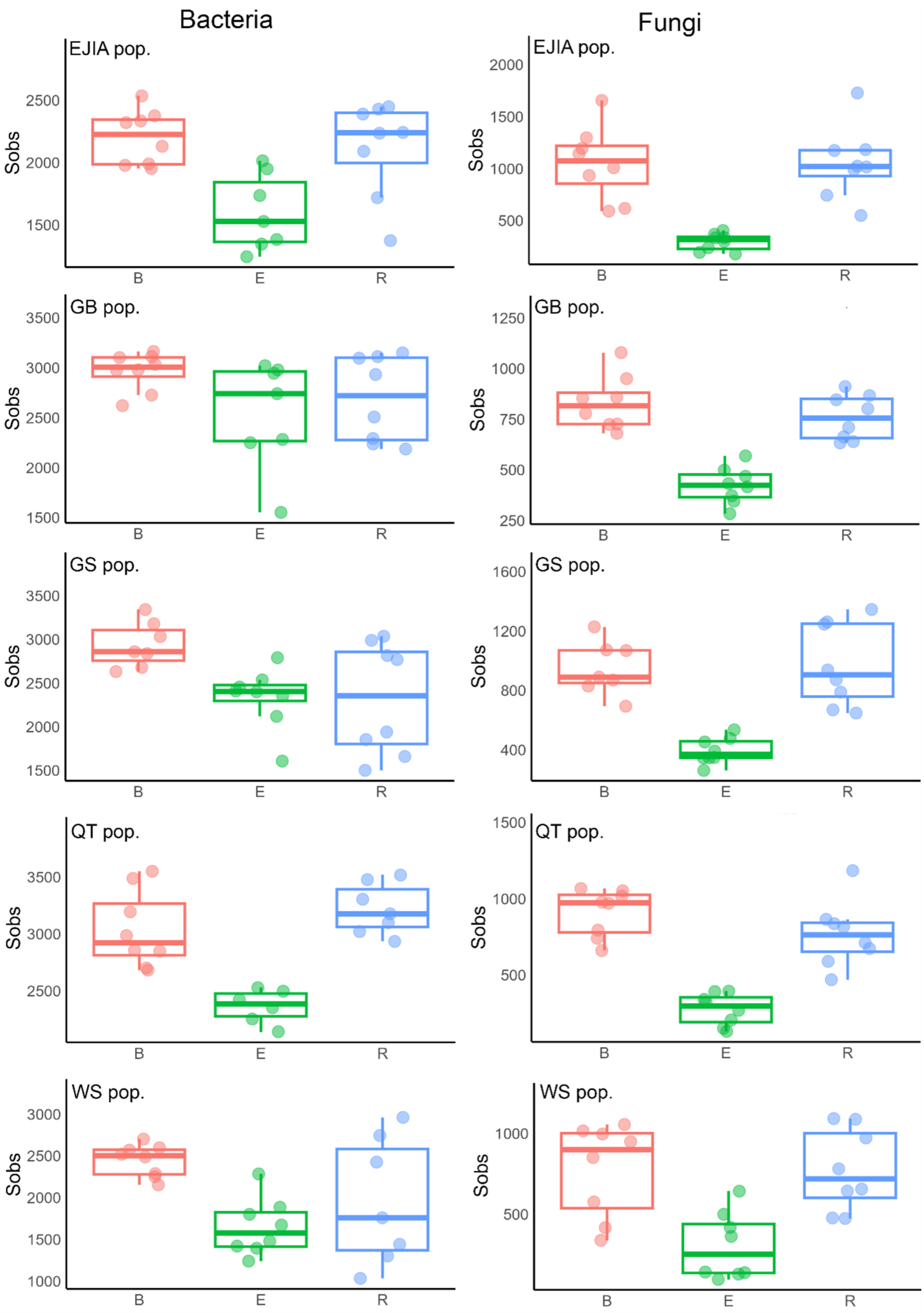

Figure S8 Species richness (Sobs) of three compartments within populations.

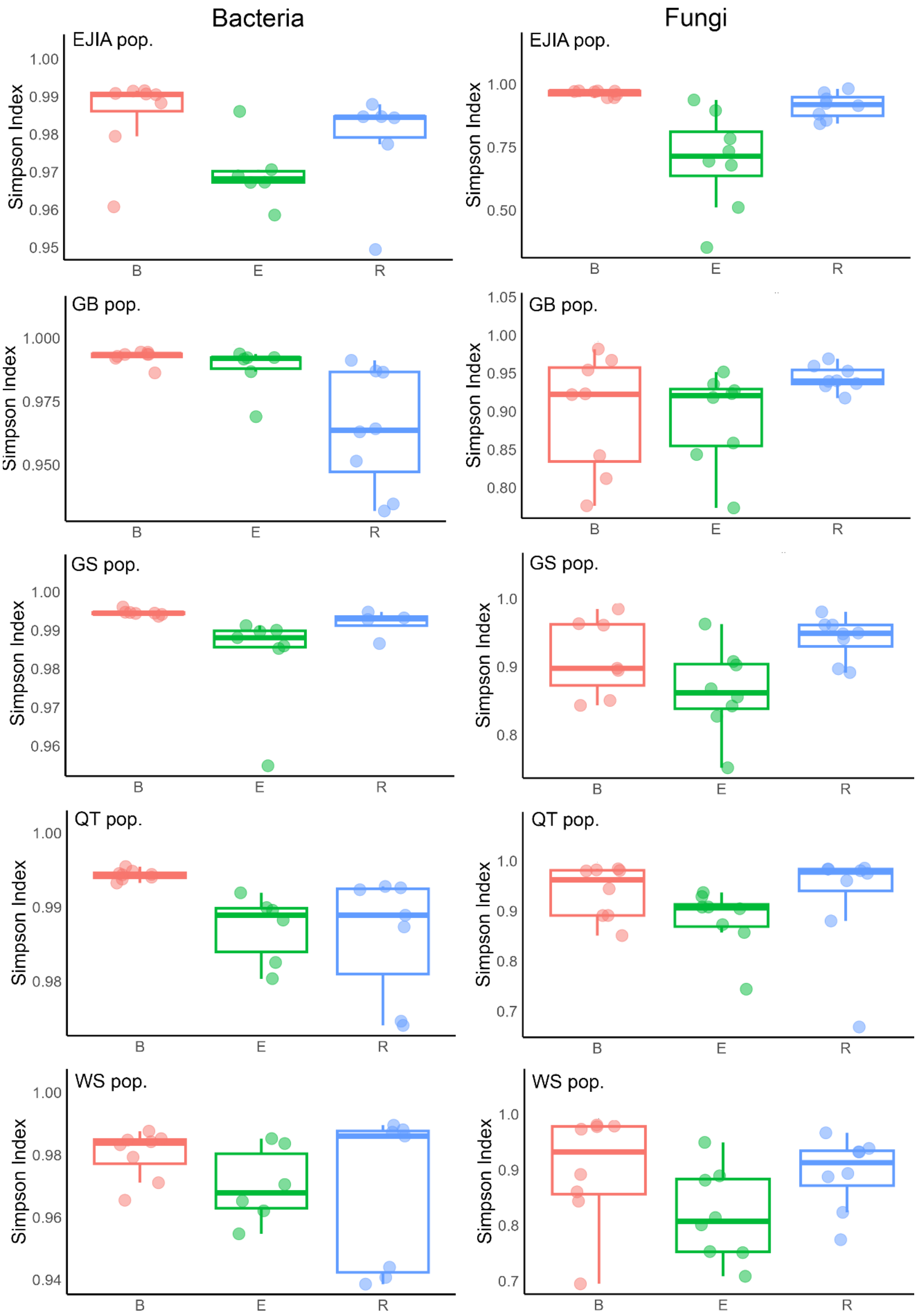

Figure S9 Species evenness (Simpson) of three compartments within populations.

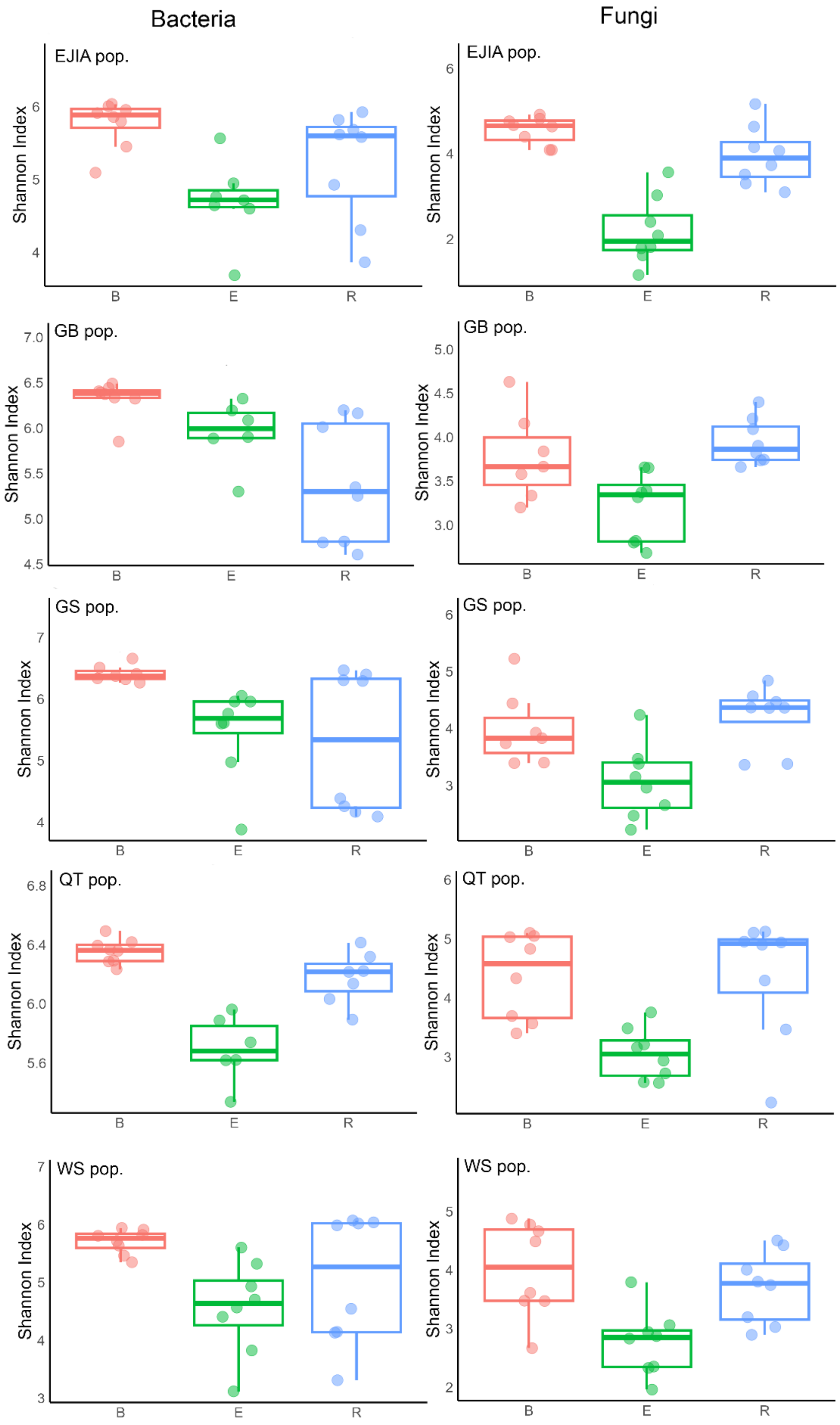

Figure S10 Species diversity (Shannon) of three compartments within populations.

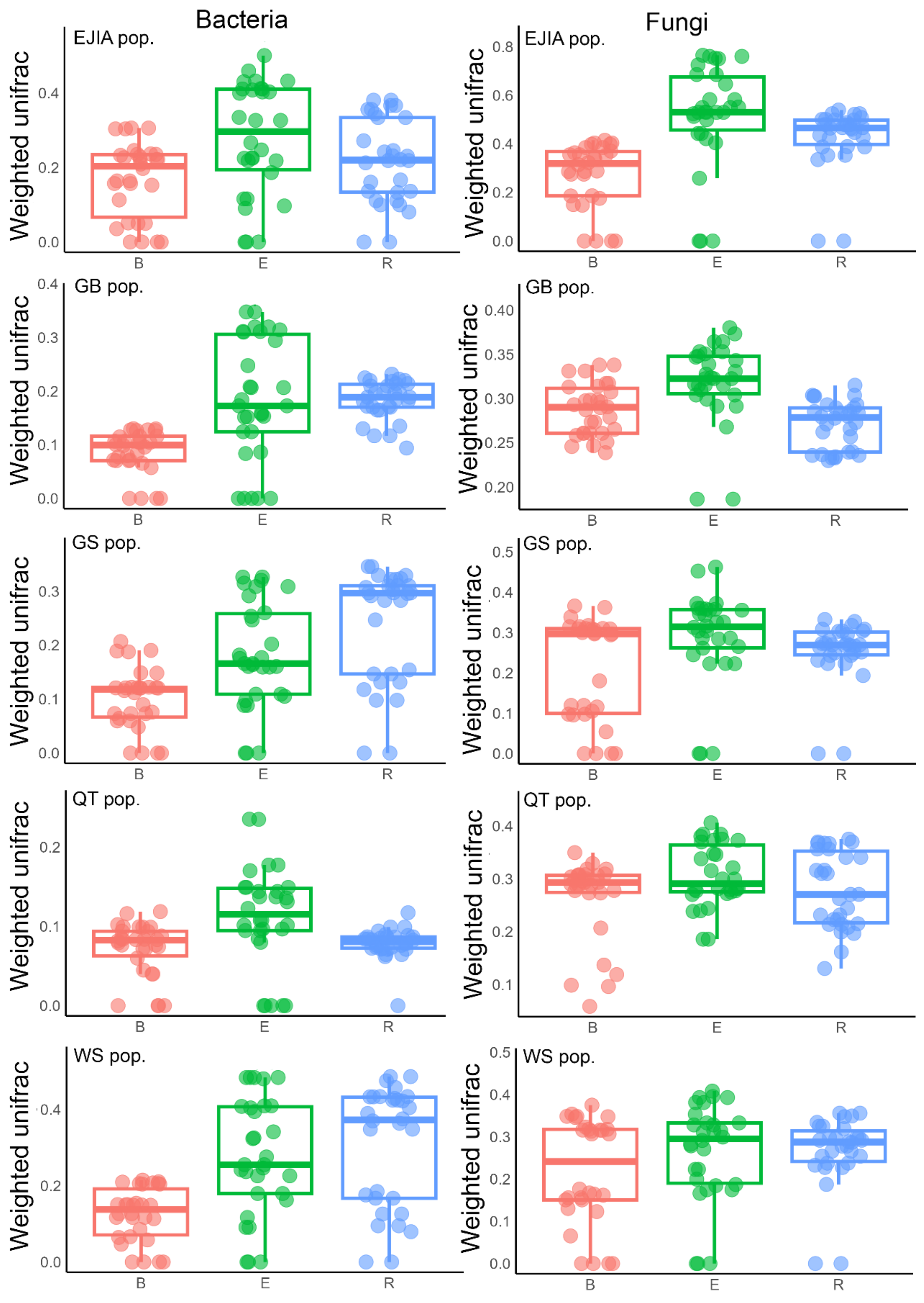

Figure S11 Beta diversity (Weighted unifrac) of three compartments within populations.

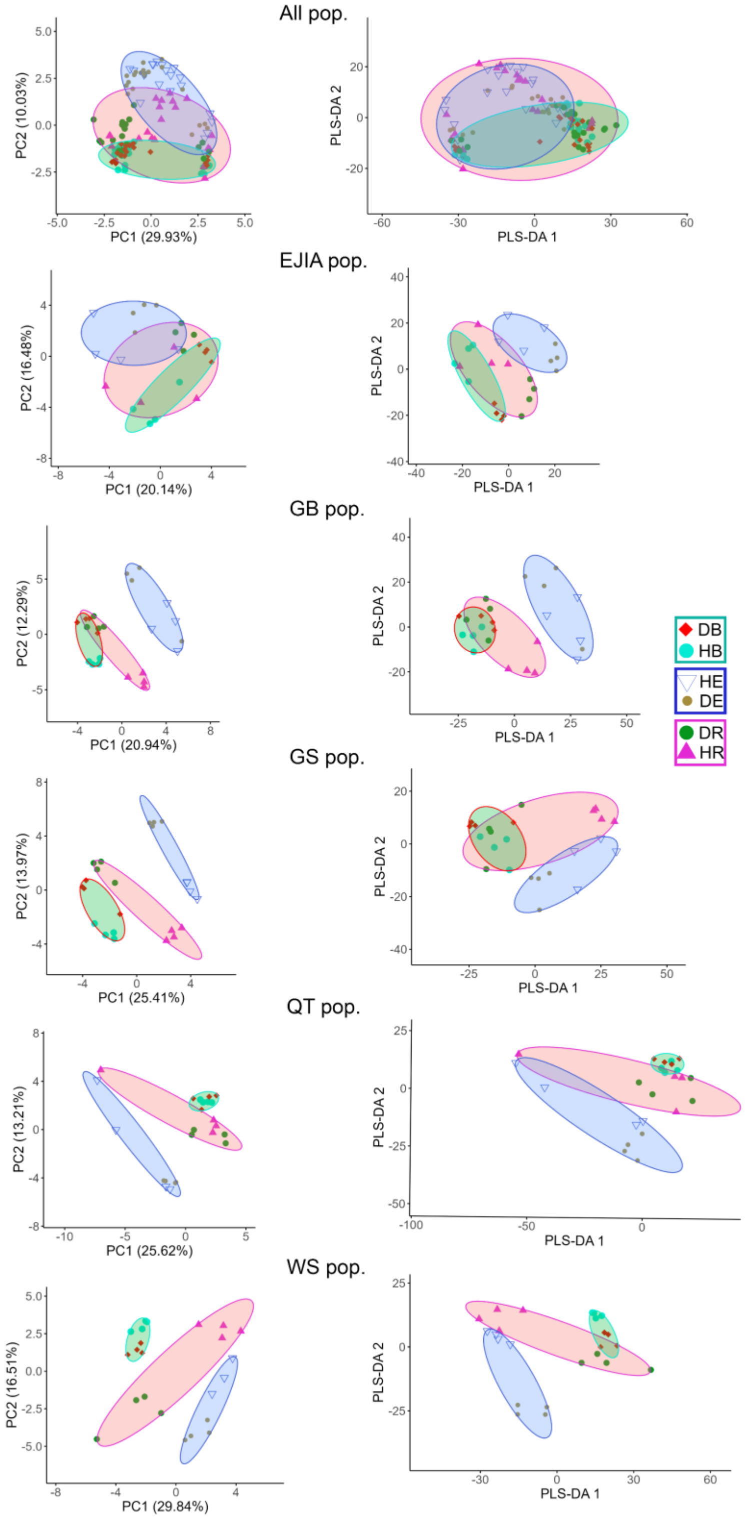

Figure S12 PCA and PLS-DA analyses of bacterial compositions in hydrated and dehydrated states of three compartments.

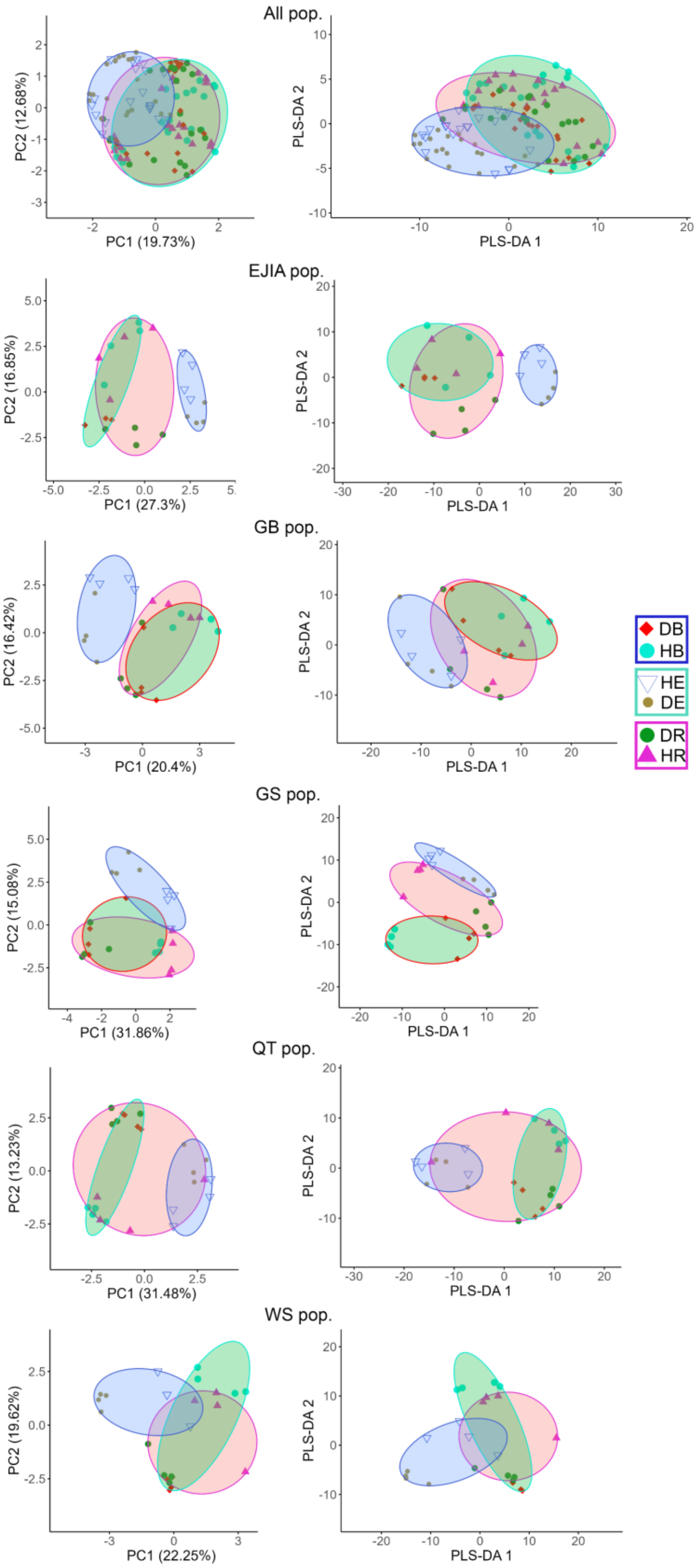

Figure S13 PCA and PLS-DA analyses of fungal compositions in hydrated and dehydrated states of three compartments.

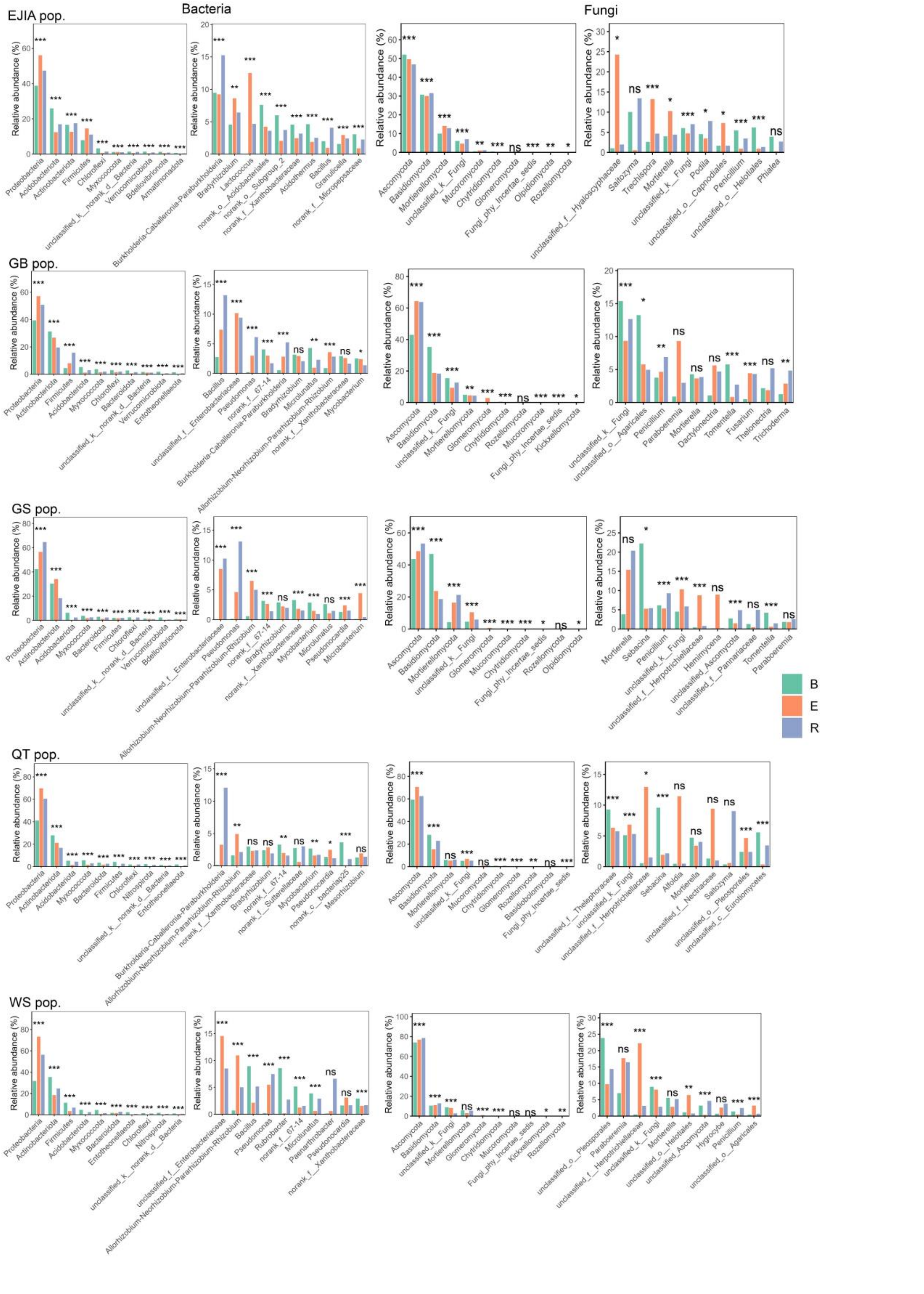

Figure S14 Differences in bacterial and fungal phyla and genera abundance across populations.

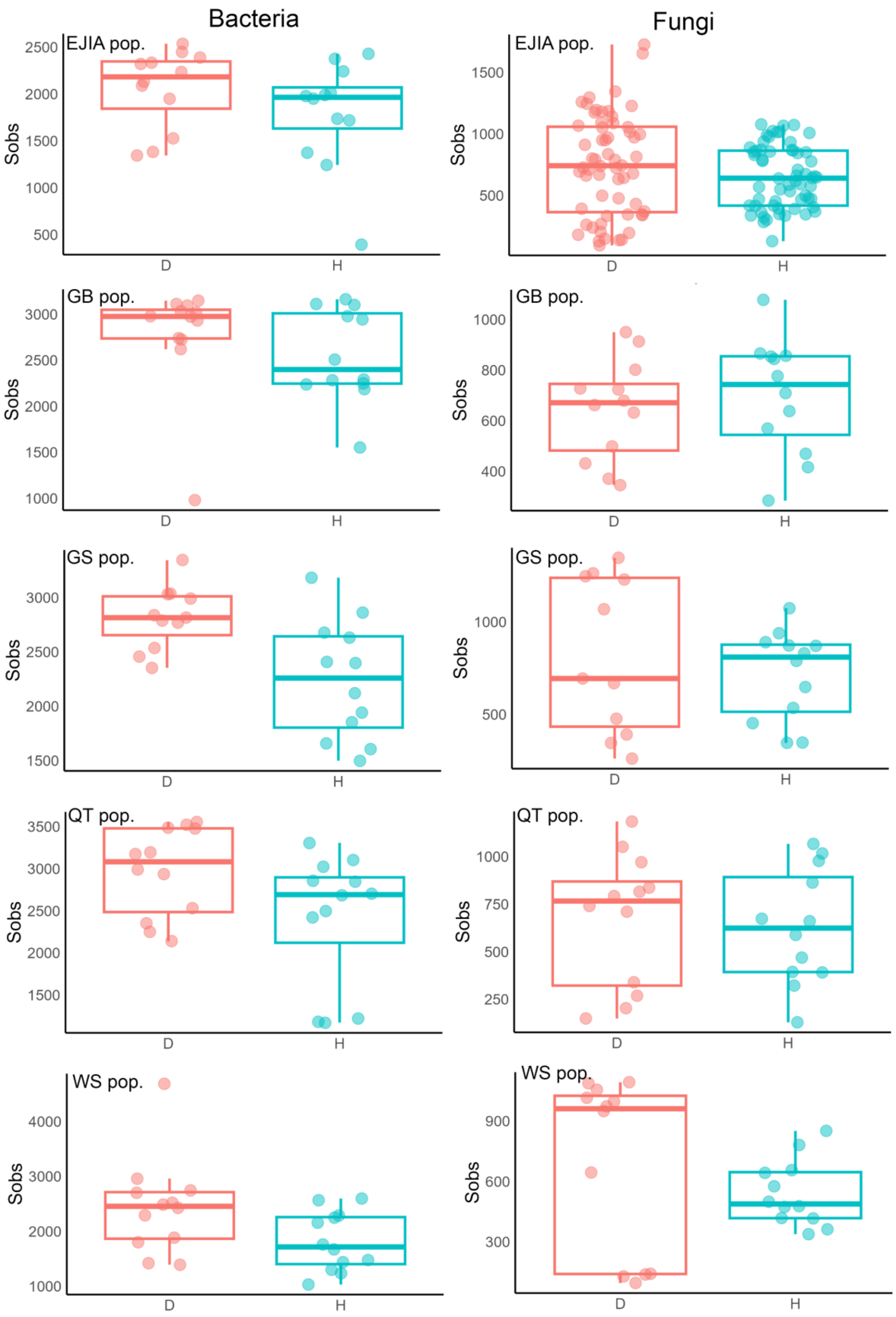

Figure S15 Sobs indices of bacterial and fungal communities in dehydrated and hydrated states.

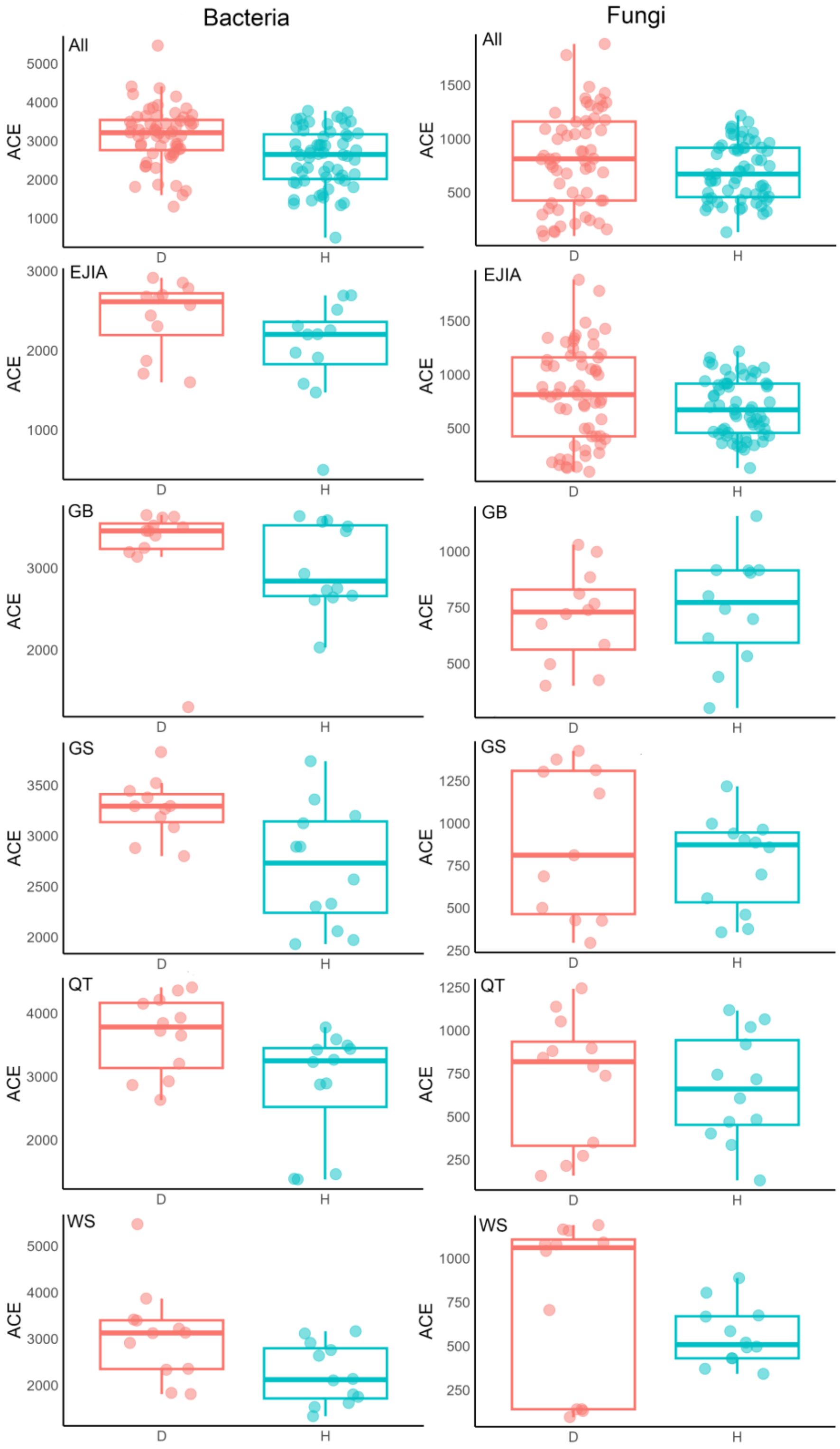

Figure S16 ACE indices of bacterial and fungal communities in dehydrated and hydrated states.

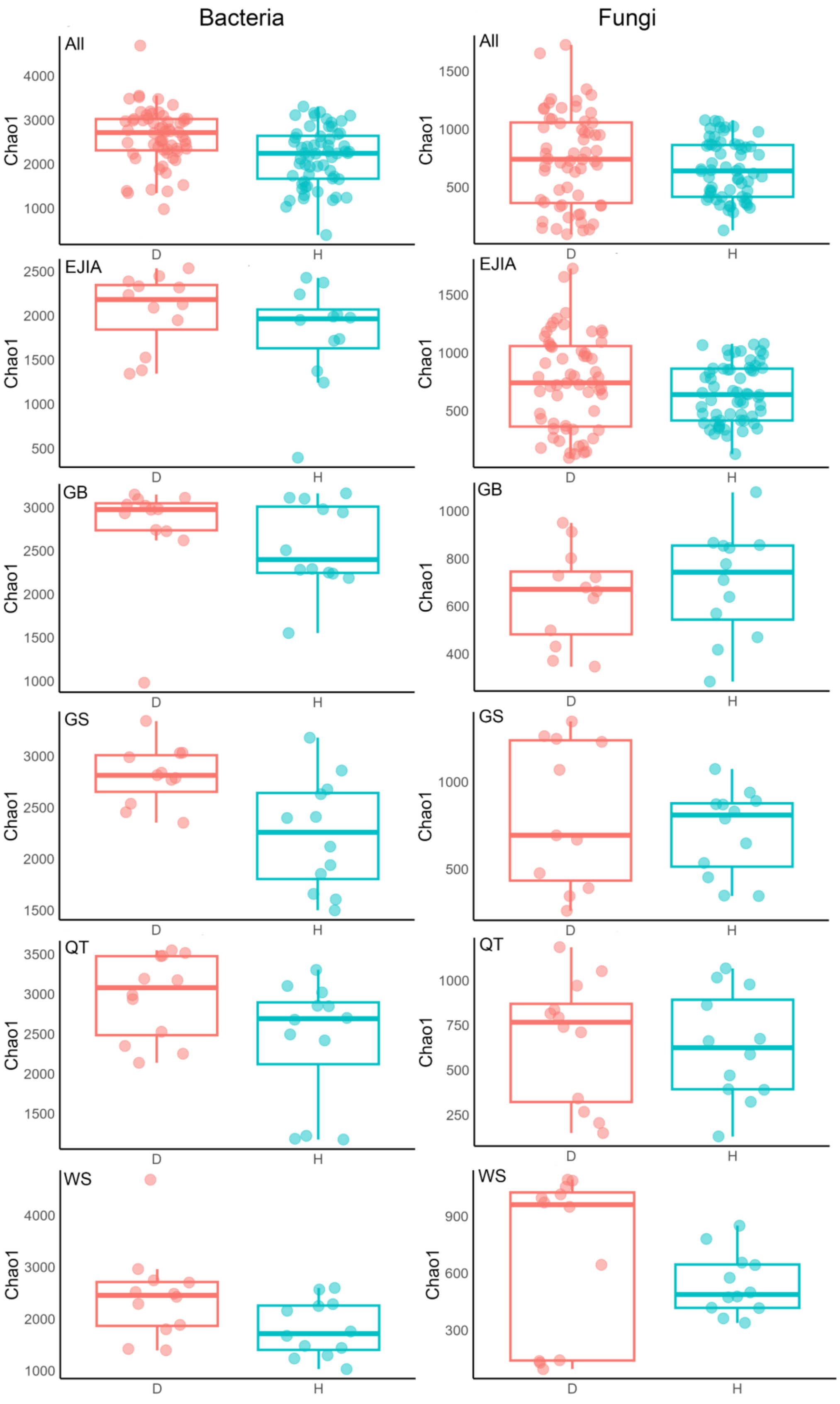

Figure S17 Chao indices of bacterial and fungal communities in dehydrated and hydrated states.

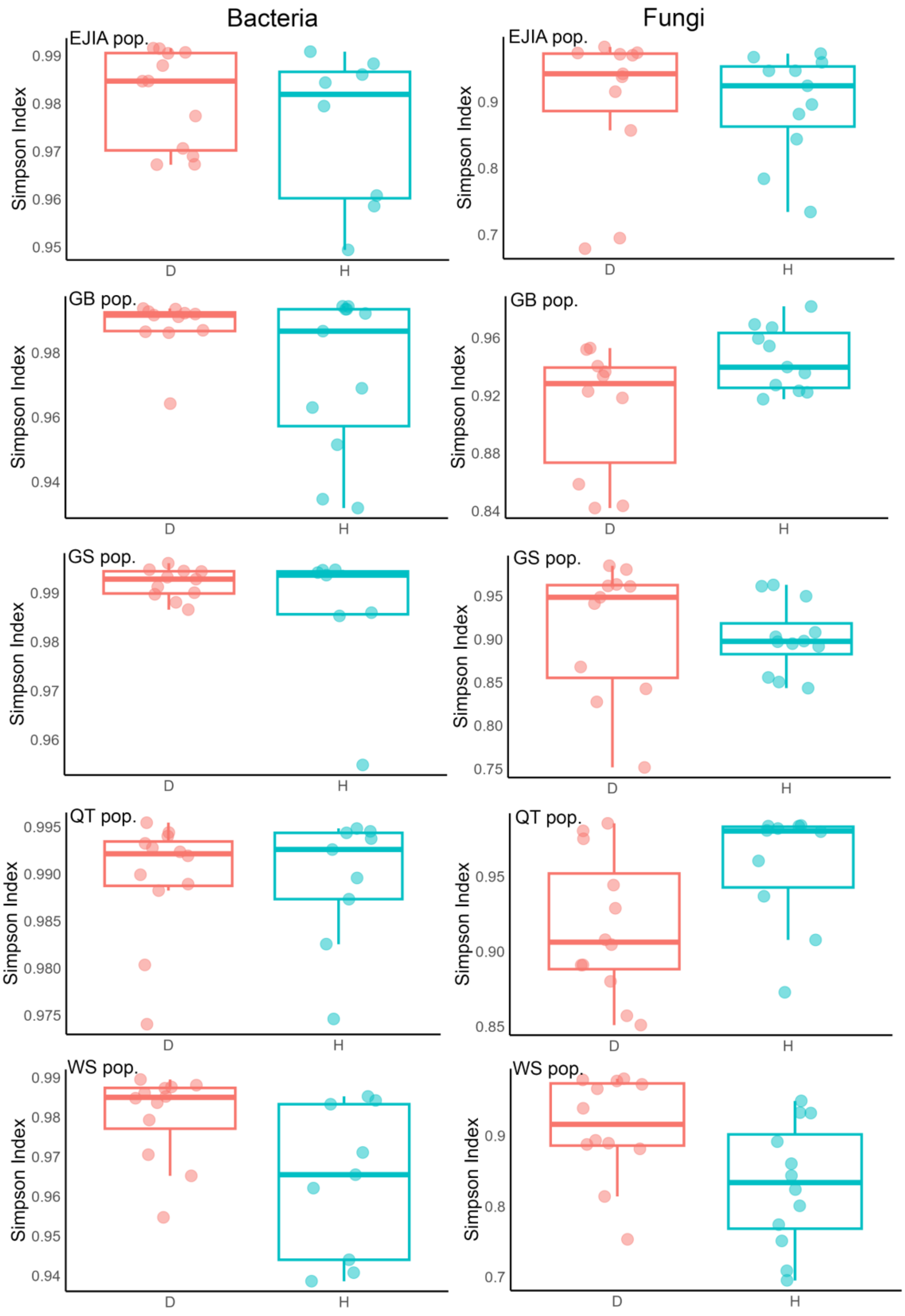

Figure S18 Simpson indices of bacterial and fungal communities in dehydrated and hydrated states.

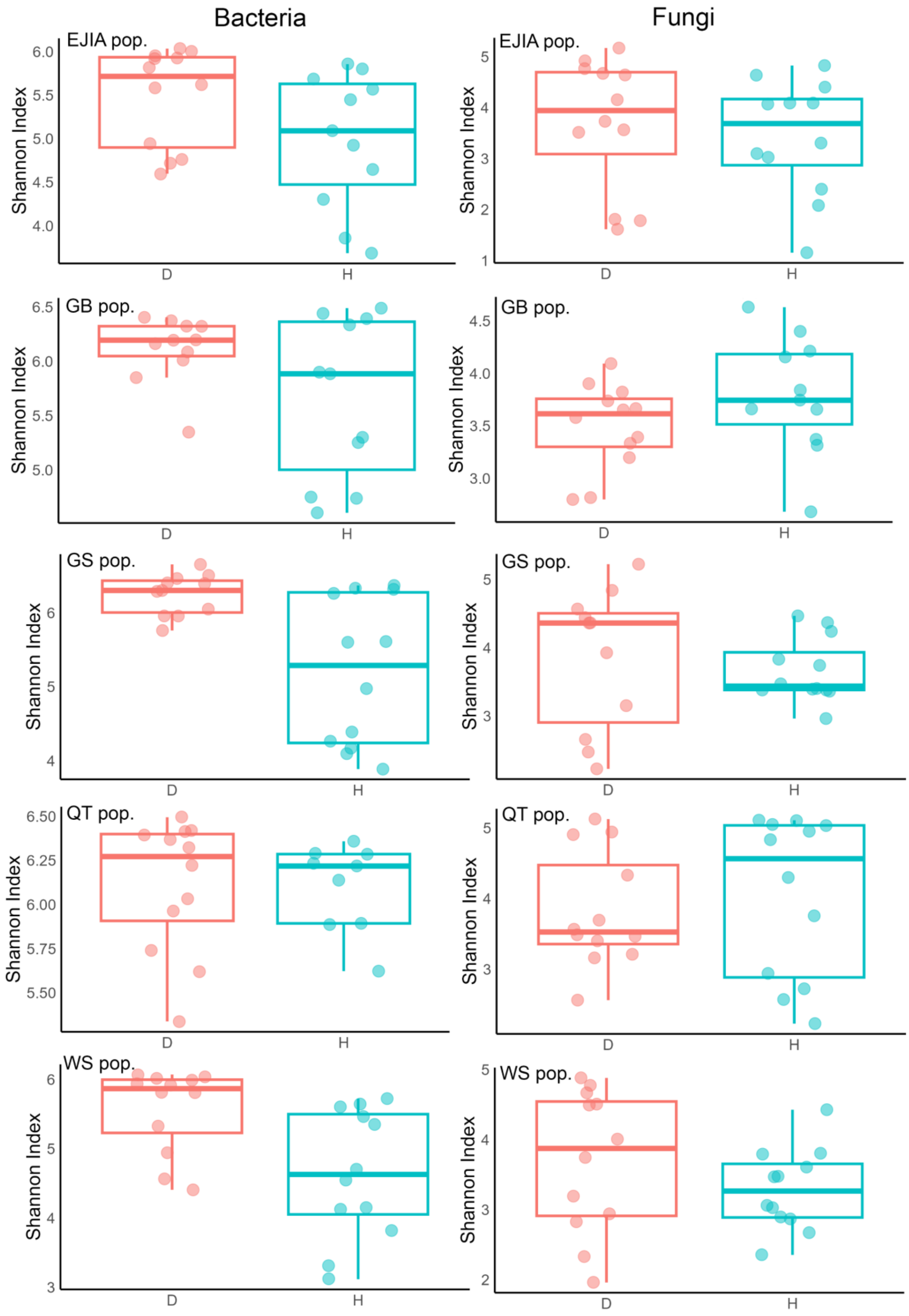

Figure S19 Shannon indices of bacterial and fungal communities in dehydrated and hydrated states.

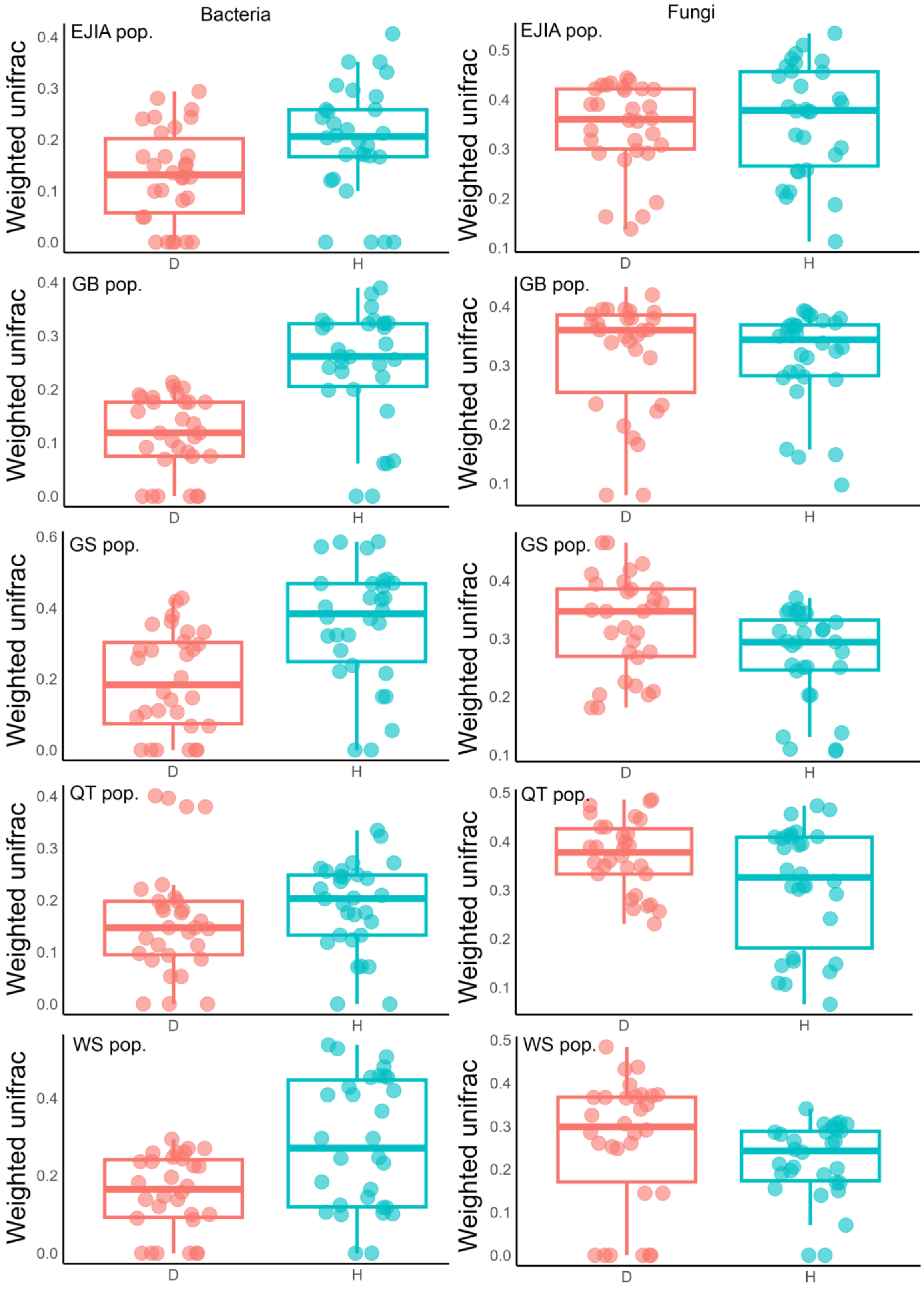

Figure S20 Beta diversity of bacterial and fungal communities based on weighted UniFrac distance in dehydrated and hydrated states.

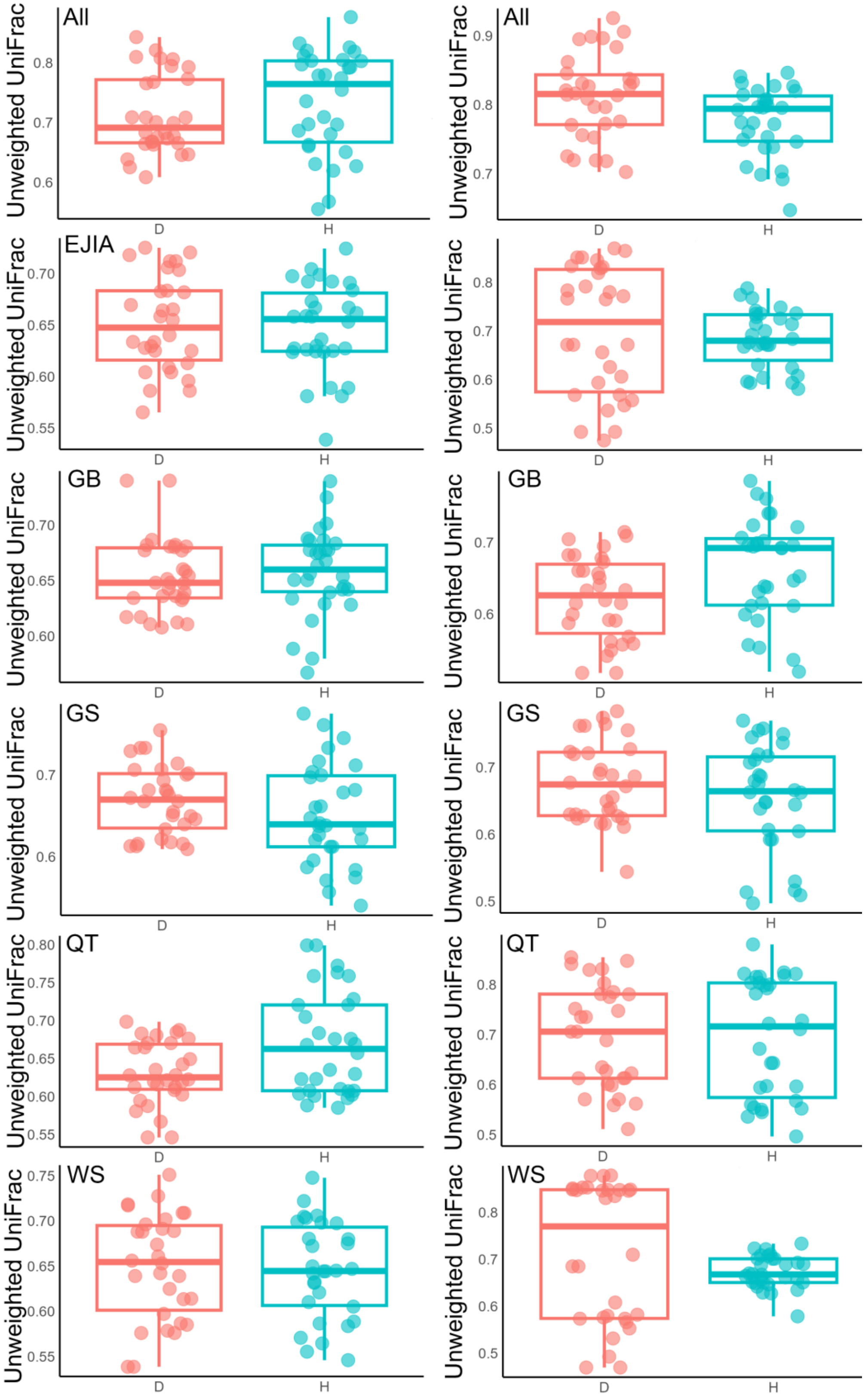

Figure S21 Beta diversity of bacterial and fungal communities based on weighted UniFrac distance in dehydrated and hydrated states.

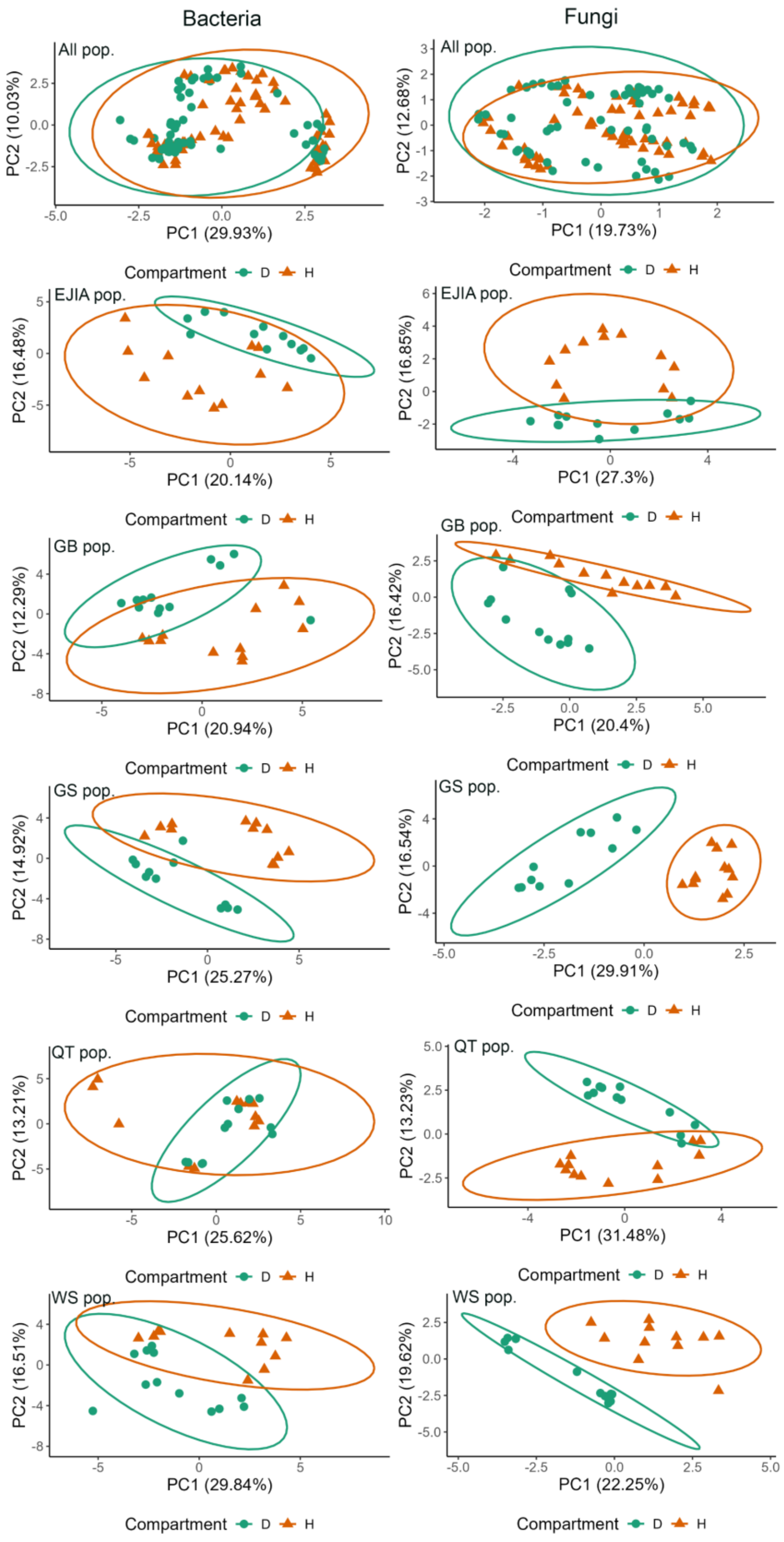

Figure S22 PCA analyses of bacterial and fungal compositions in hydrated and dehydrated states of compartments.

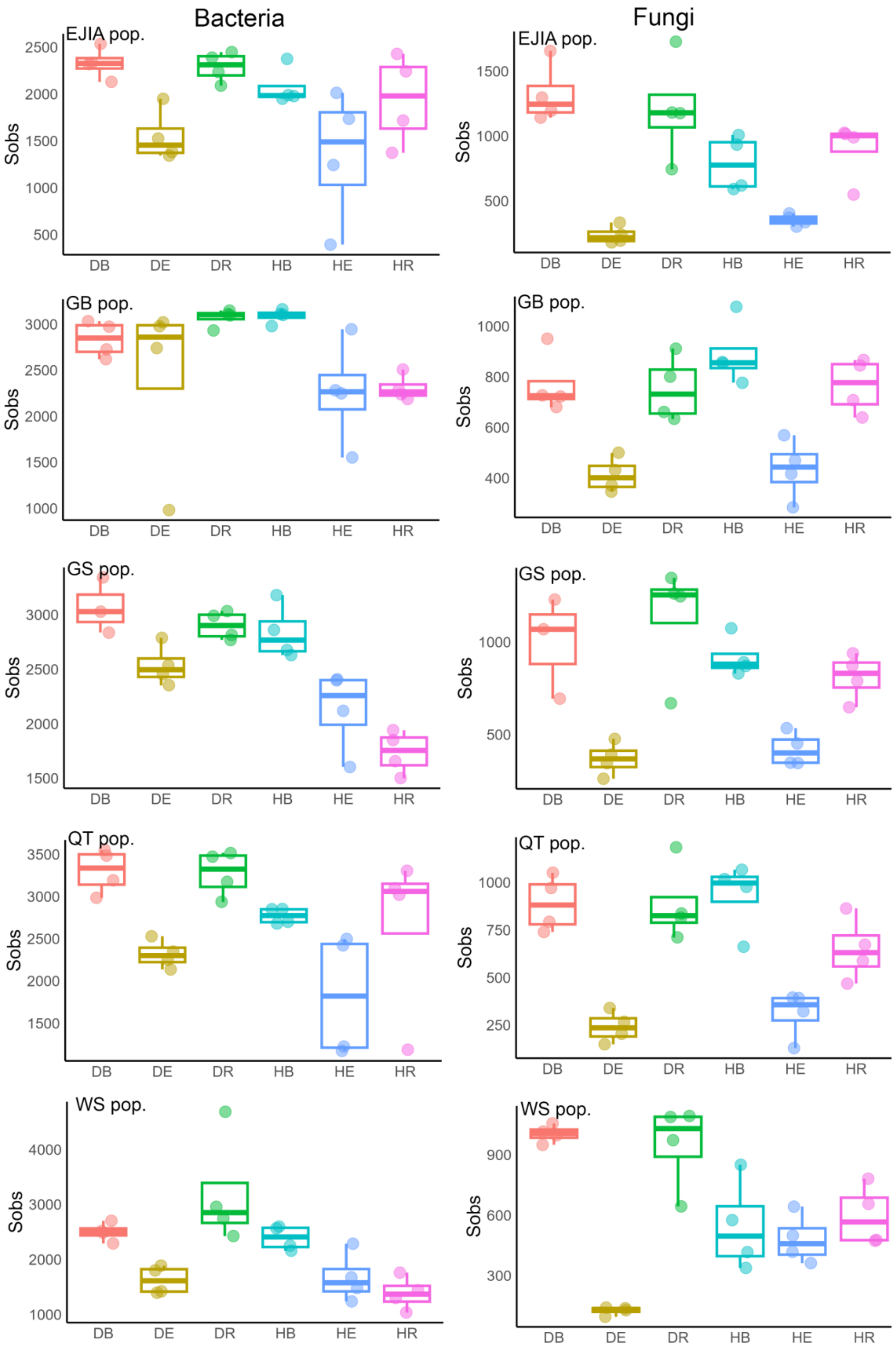

Figure S23 alpha diversity of bacterial and fungal communities across six compartments in dehydrated and hydrated states.

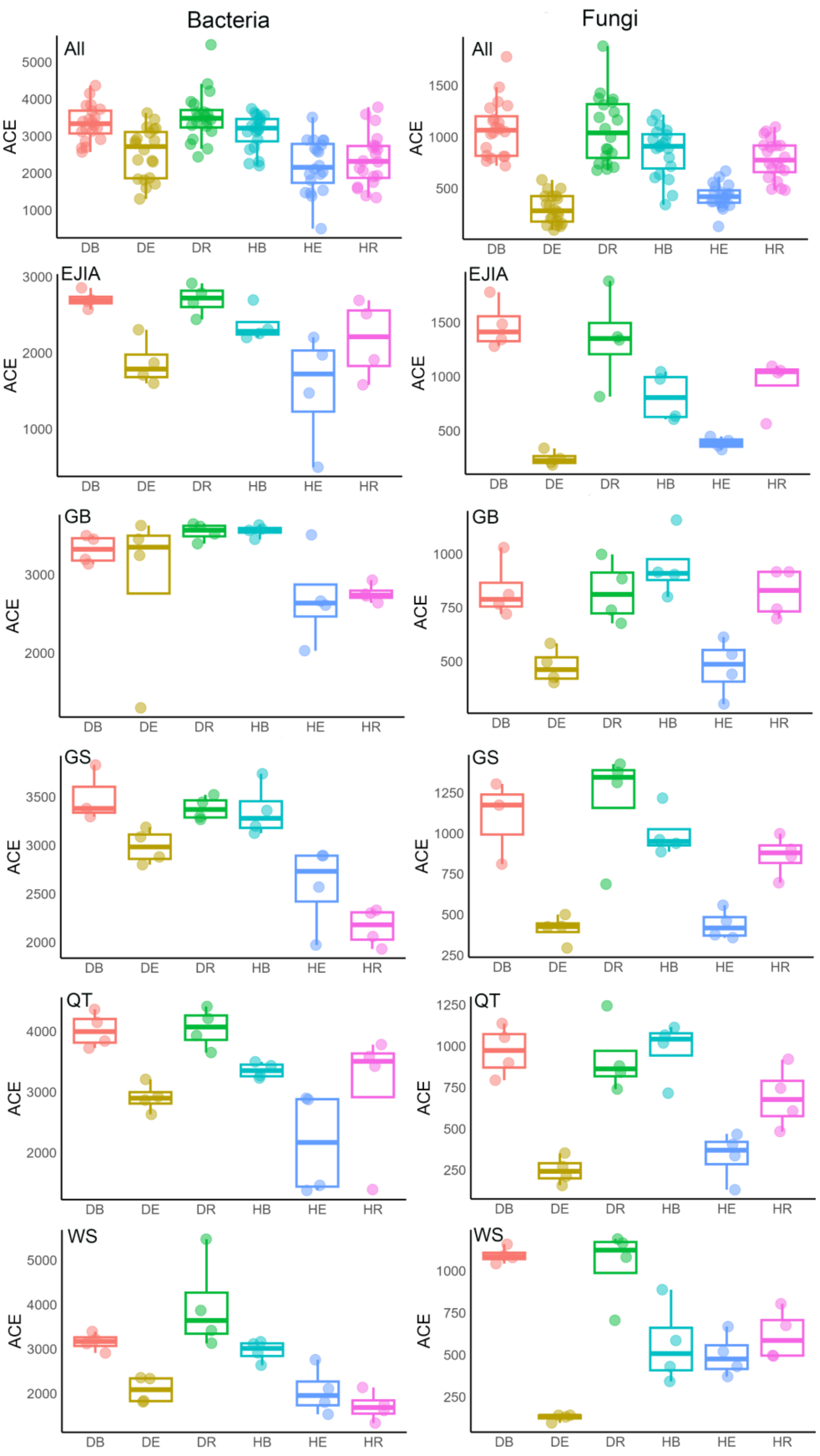

Figure S24 ACE indices of bacterial and fungal communities in dehydrated and hydrated states.

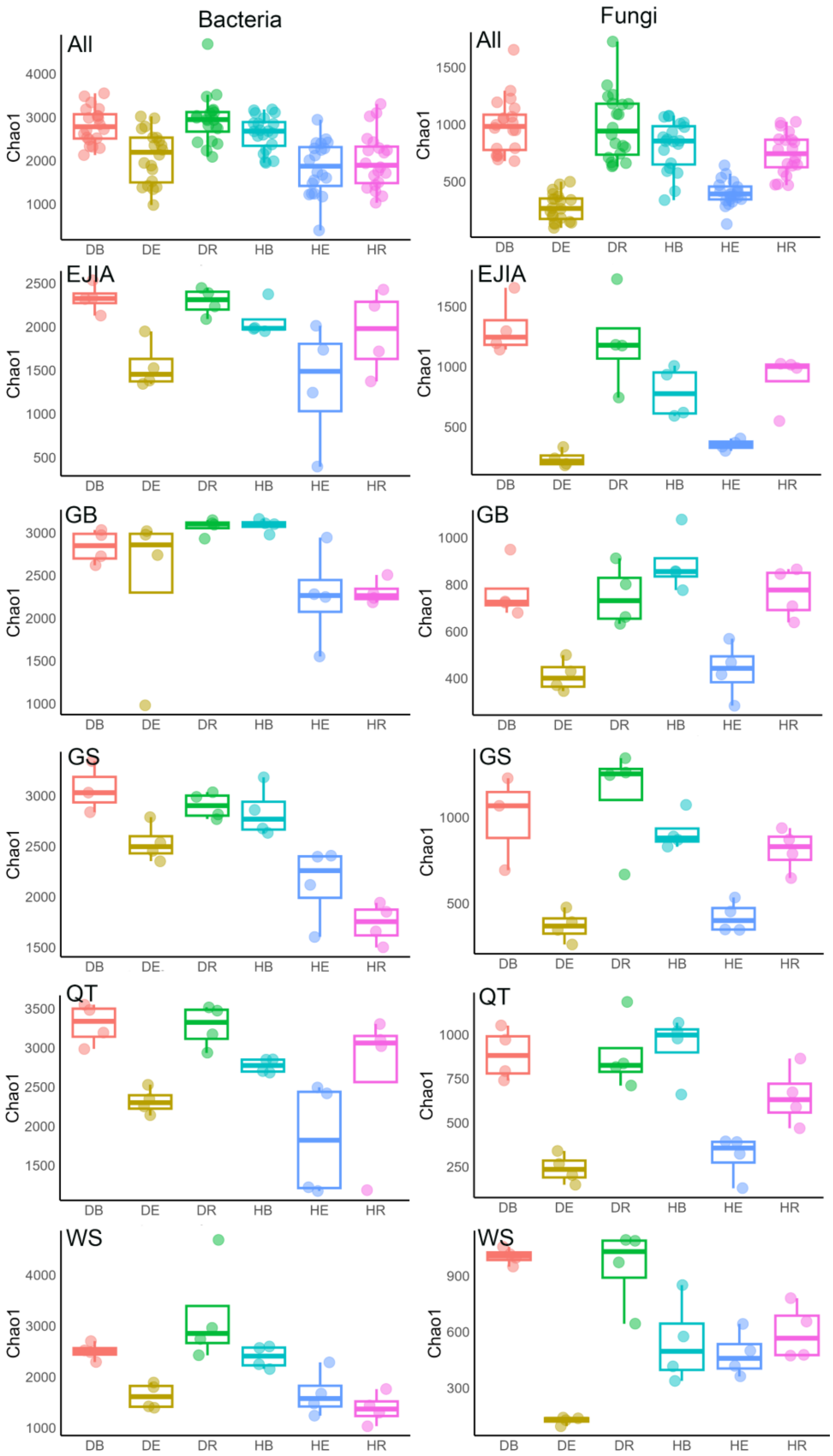

Figure S25 Chao indices of bacterial and fungal communities in dehydrated and hydrated states.

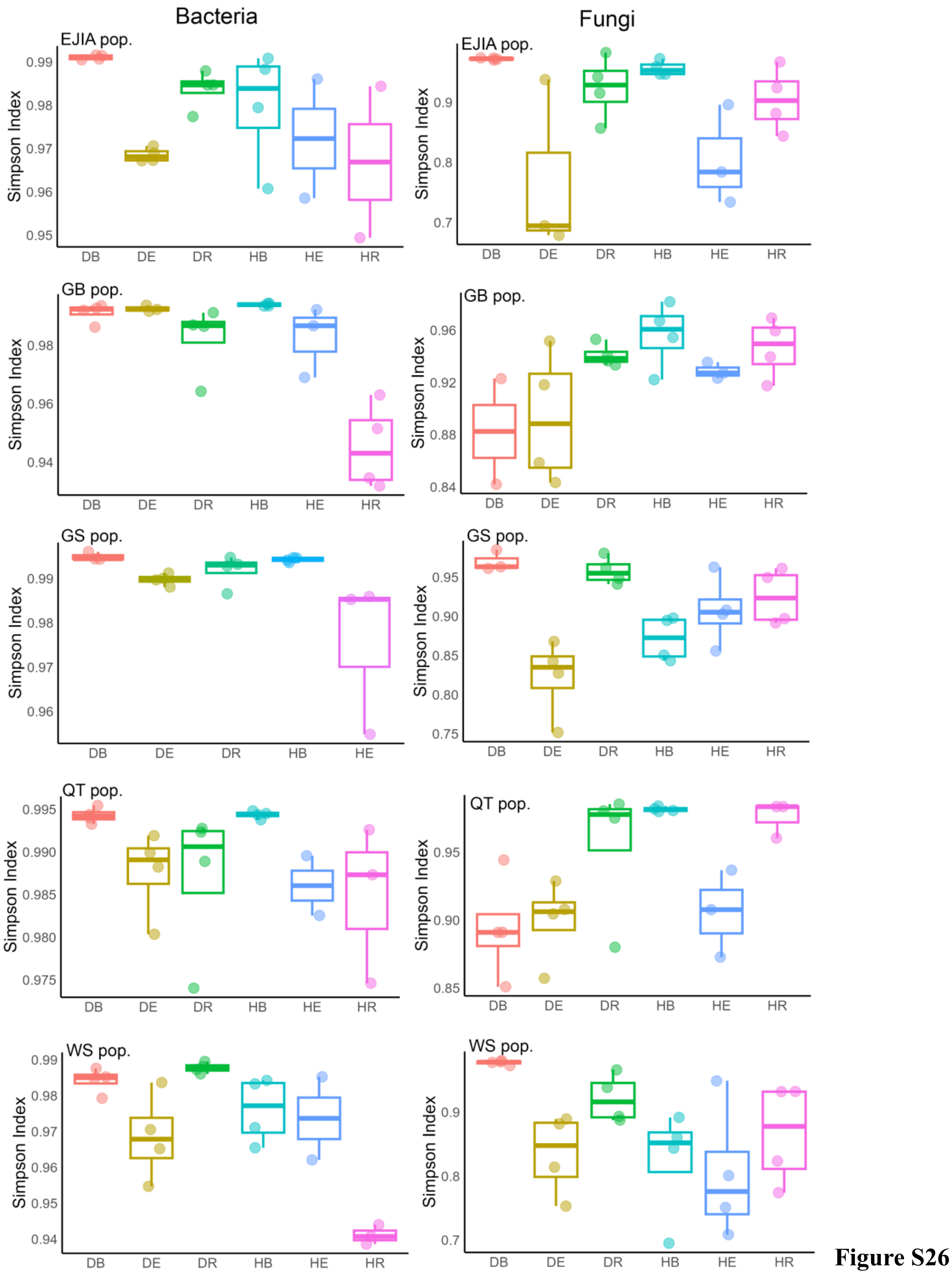

Figure S26 Simpson indices of bacterial and fungal communities in dehydrated and hydrated states.

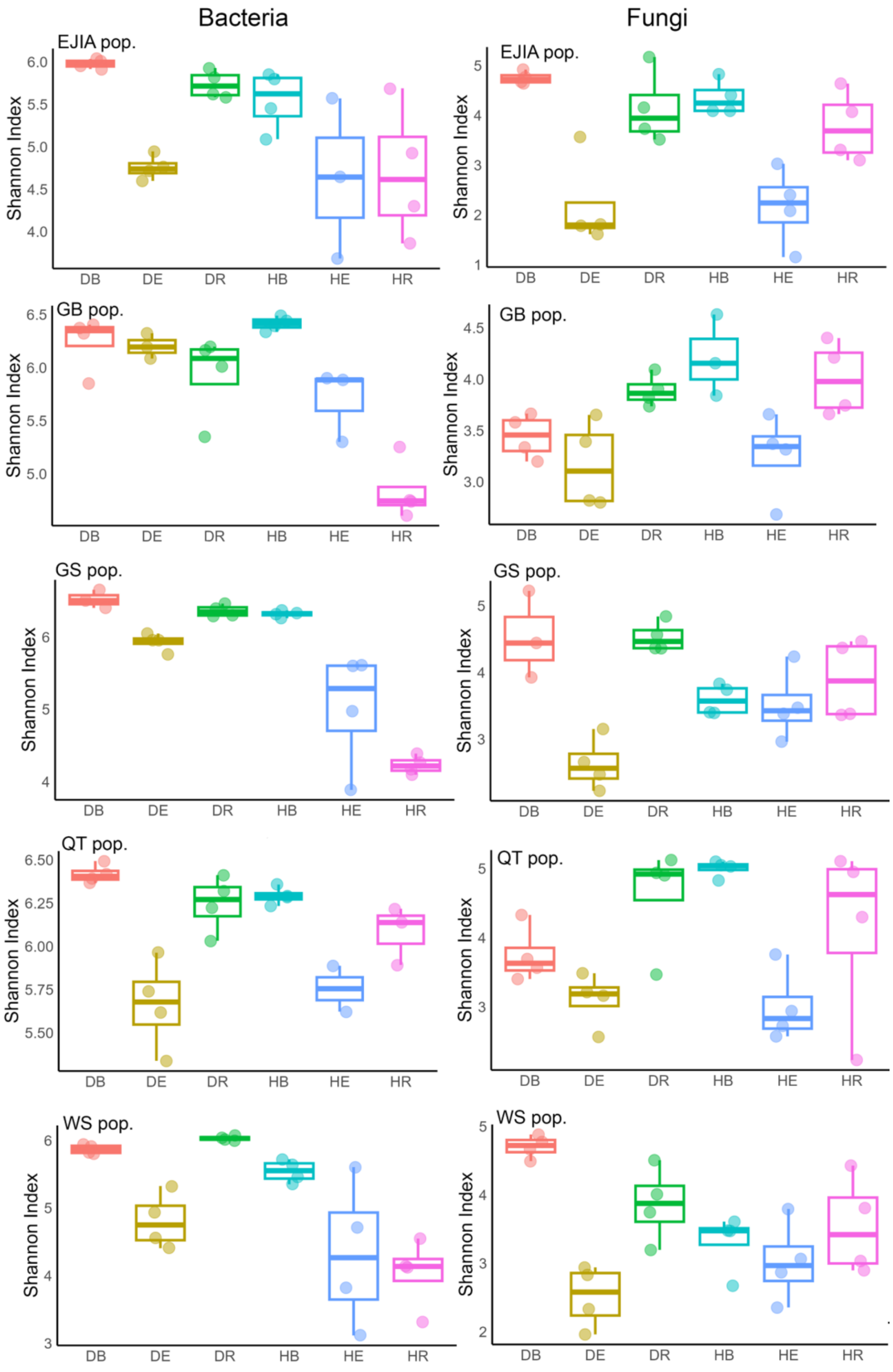

Figure S27 Simpson indices of bacterial and fungal communities in dehydrated and hydrated states.

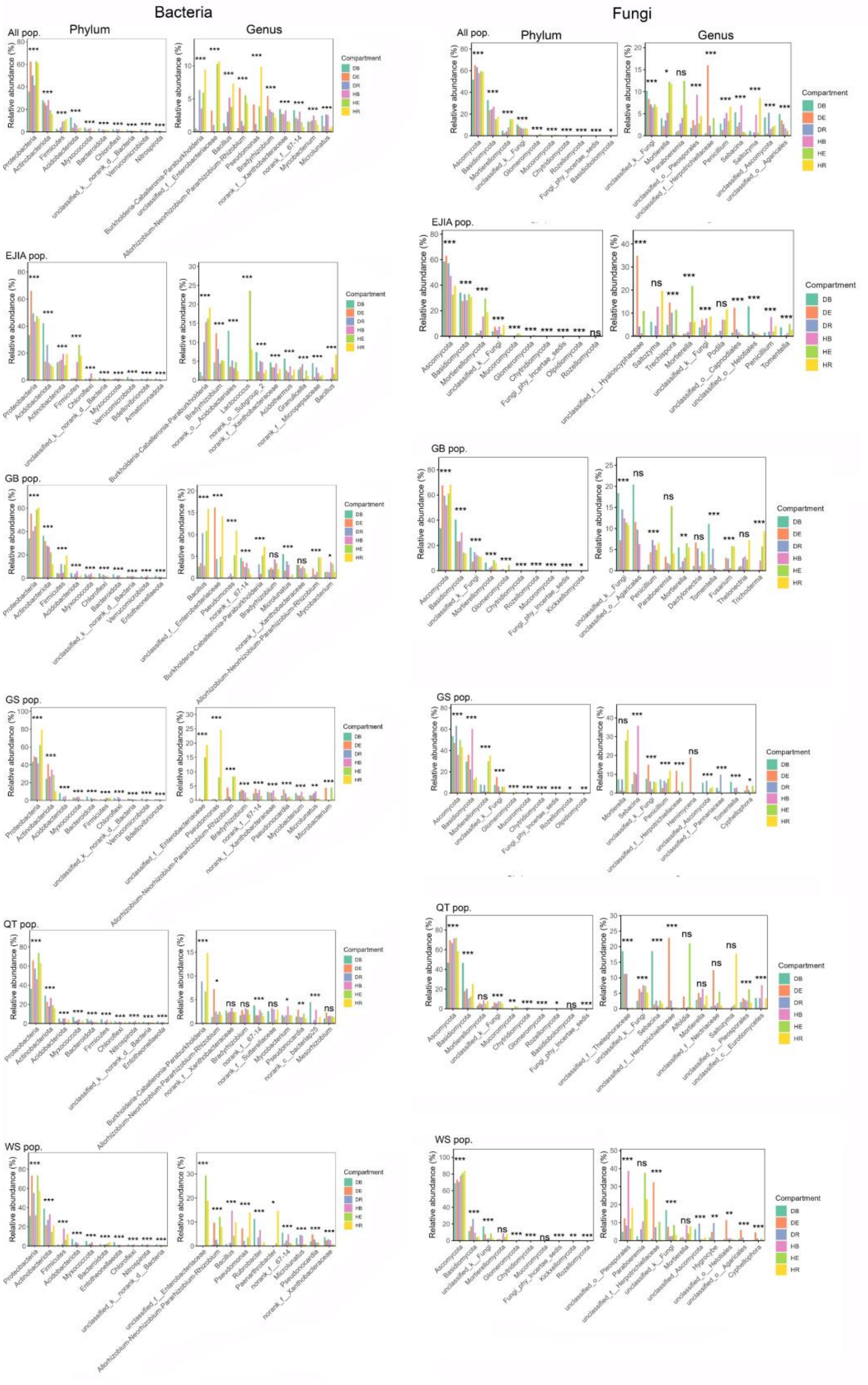

Figure S28 differential abundance of bacterial and fungal communities in 6 compartments of D and H groups.

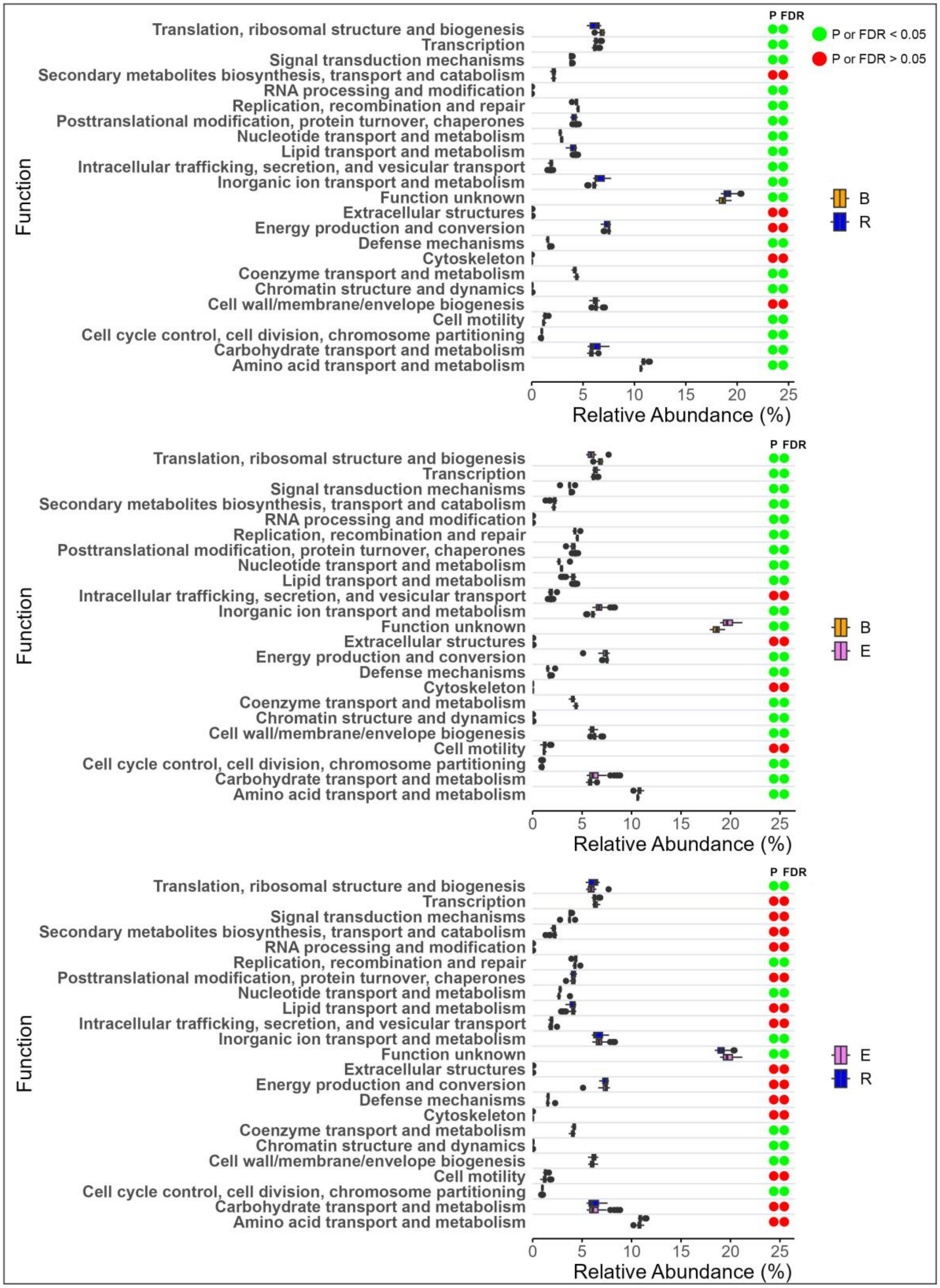

Figure S29 Differences in the abundance of COG annotations at level 2 among bacterial communities across adjacent compartments (B vs. R, B vs. E, and E vs. R), where B represents bulk soil, R denotes rhizosphere soil, and E stands for root endosphere. P and FDR values show whether the functions between two compartments are significantly different or not.

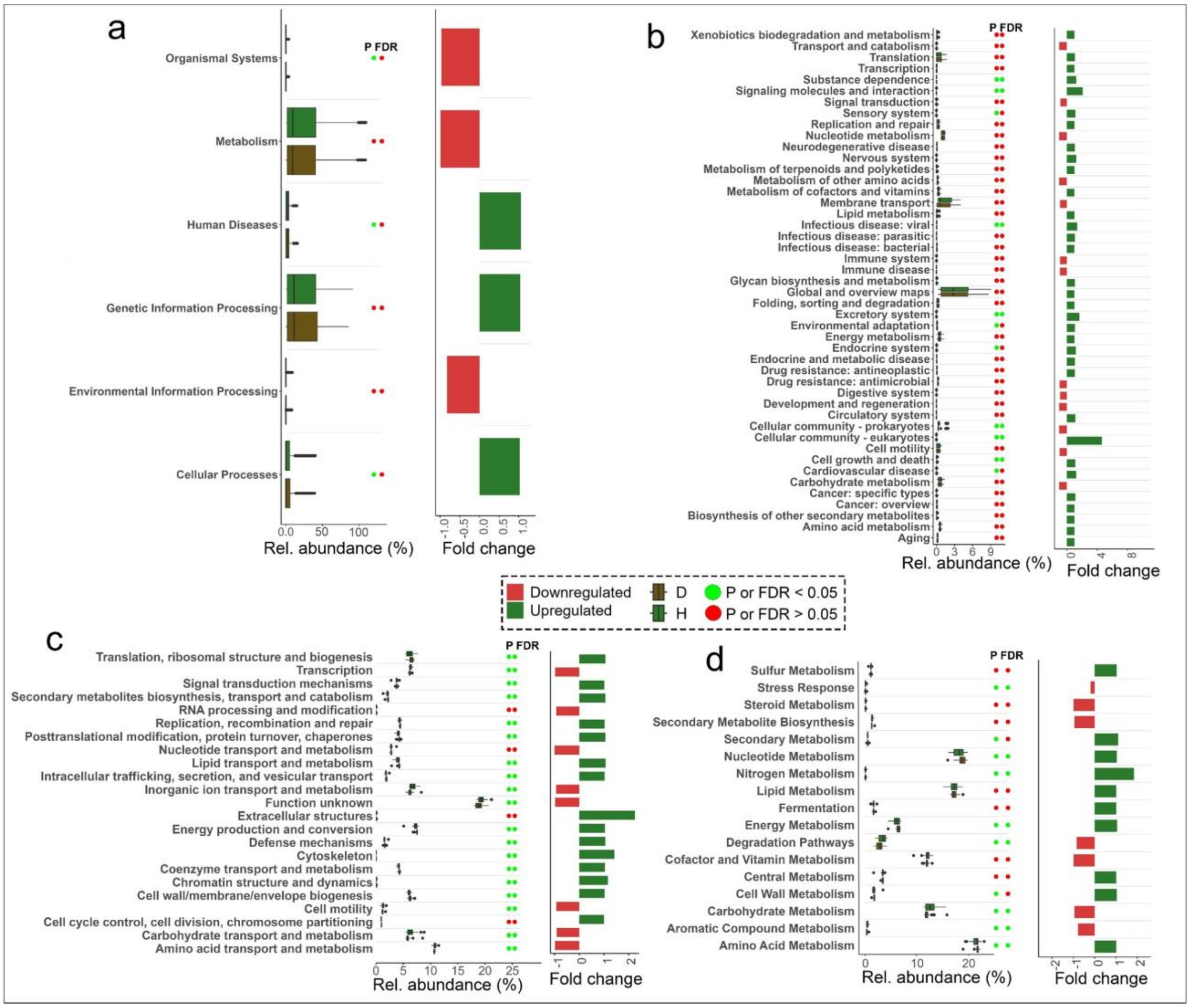

Figure S30 Functional differences of the bacterial communities between hydrated (H) and dehydrated (D) groups. a, b Level 1 (a) and 2 (b) of KEGG annotations. c, d, level 2 of COG (c) and MetaCyc (d) annotations, respectively. Fold change panels show whether the functions are upregulated (when the function increases during drought) or downregulated (when function decreases during drought). P and FDR values show the significance of the fold change.

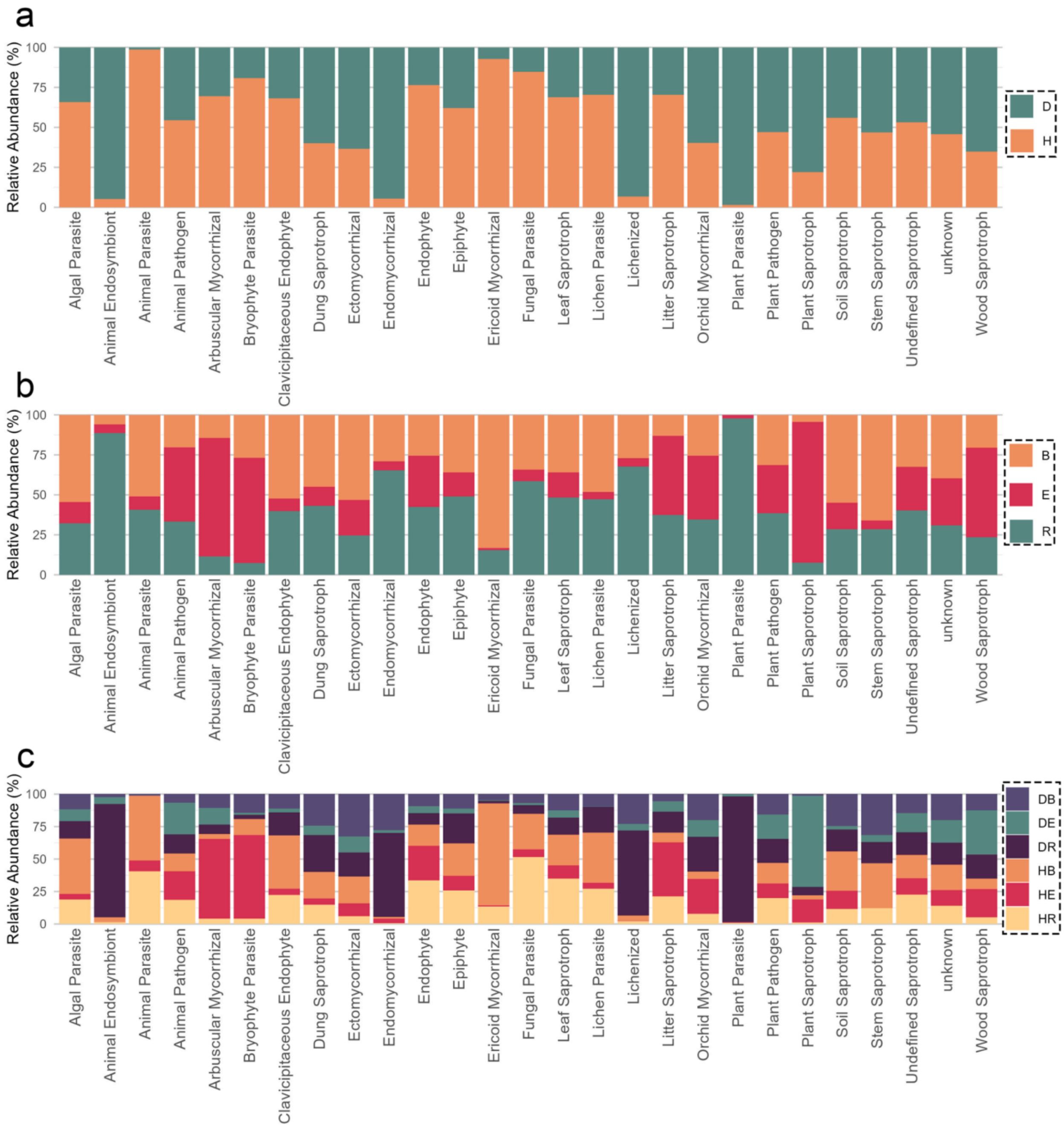

Figure S31 Ecological roles of fungal communities. (a) Relative abundance of the functions between hydrated (H) and dehydrated (D) samples. (b) Relative abundance of the functions bulk soil (B), rhizosphere soil (R) and root endosphere (E) for all hydrated and dehydrated samples. Relative abundance of the functions for 3 compartments under both hydrated and dehydrated conditions, DB: Dehydrated bulk soil, DE: Dehydrated root endosphere, DR: Dehydrated rhizosphere, HB: Hydrated bulk soil, HE: Hydrated root endosphere and HR: Hydrated rhizosphere .
